# Beclin 1 functions as a negative modulator of MLKL oligomerisation by integrating into the necrosome complex

**DOI:** 10.1038/s41418-020-0561-9

**Published:** 2020-05-26

**Authors:** Jinho Seo, Daehyeon Seong, Young Woo Nam, Chi Hyun Hwang, Seung Ri Lee, Choong-Sil Lee, Young Jin, Han-Woong Lee, Doo-Byoung Oh, Peter Vandenabeele, Jaewhan Song

**Affiliations:** 1 Department of Biochemistry, College of Life science and Biotechnology, Yonsei University, Seoul, Korea; 2 Environmental Disease Research Center, Korea Research Institute of Bioscience and Biotechnology (KRIBB), Daejeon, Korea; 3 Department of Biosystems and Bioengineering, KRIBB School of Biotechnology, University of Science and Technology (UST), Daejeon, Korea; 4 VIB-UGent Center for Inflammation Research, VIB, B-9052 Ghent, Belgium; 5 Department of Biomedical Molecular Biology, Ghent University, B-9052 Ghent, Belgium

**Keywords:** Cell biology, Molecular biology

## Abstract

Necroptosis is a form of regulated cell death caused by formation of the necrosome complex. However, the factors modulating this process and the systemic pathophysiological effects of necroptosis are yet to be understood. Here, we identified that Beclin 1 functions as an anti-necroptosis factor by being recruited into the necrosome complex upon treatment with TNFα, Smac mimetic, and pan-caspase inhibitor and by repressing MLKL oligomerisation, thus preventing the disruption of the plasma membrane. Cells ablated or knocked-out for Beclin 1 become sensitised to necroptosis in an autophagy-independent manner without affecting the necrosome formation itself. Interestingly, the recruitment of Beclin 1 into the necrosome complex is dependent on the activation and phosphorylation of MLKL. Biochemically, the coiled-coil domain (CCD) of Beclin 1 binds to the CCD of MLKL, which restrains the oligomerisation of phosphorylated MLKL. Finally, Beclin 1 depletion was found to promote necroptosis in leukaemia cells and enhance regression of xenografted-tumour upon treatment with Smac mimetics and caspase inhibitors. These results suggest that Beclin 1 functions as a negative regulator in the execution of necroptosis by suppressing MLKL oligomerisation.

## Introduction

Necroptosis is a programmed cell death mechanism, initiated by various cytokine, death receptors and pattern recognizing receptors (PRR) when caspase-8 is inhibited by pharmacological treatment or genetic modification [1]. The binding of the respective ligand induces the recruitment of RIPK Homotypic Interaction Motif (RHIM) containing proteins, subsequently leading the recruitment and activation of the RHIM domain containing RIPK3 [2–7]. In the necrosome complex, RIPK1 and RIPK3 are activated by autophosphorylation and the recruited MLKL is subsequently phosphorylated by RIPK3, resulting in MLKL oligomerisation through the interaction of its coiled-coil domains (CCD) [8–10]. Oligomerised MLKL is translocated onto the plasma membrane, consequently executing necroptosis via plasma membrane permeabilization and regulation of ion channels [8, 9, 11–13].

Checkpoints for executing necroptosis have been extensively studied due to the importance of necroptosis in many inflammatory, degenerative and infectious diseases [14]. In general, RIPK1 as a scaffold promotes cell survival signalling while its kinase activity is implicated in apoptotic and necroptotic cell death. Induction of RIPK1 kinase activity-mediated cell death is tightly controlled by several checkpoints at different levels. RIPK1 ubiquitination by cIAPs or the linear ubiquitin chain assembly complex is an early checkpoint, which stabilizes complex I formation leading to NF-κB and MAPK mediated gene induction and survival signalling, thereby resulting in the inhibition of complex II formation. Indeed, RIPK1 phosphorylation by IKKα/β, TAK1, MK2, or TBK1 upon TNF stimulation constitutes the second level of checkpoints determining cell fate, which prevents the formation of complex II [15–23]. In addition to RIPK1 modification, RIPK3 posttranslational modifications are crucial events in necroptosis. CHIP and Pellino 1 ubiquitinate and degrade RIPK3 protein levels in an ubiquitin-mediated lysosome- and proteasome-dependent manner, suppressing necroptosis [24, 25]. A20 removes RIPK3 ubiquitination, which restricts necrosome complex formation [26]. TNF receptor-associated factor 2 (TRAF2) is also an inhibitor of necrosome complex formation. TRAF2 constitutively binds to MLKL under normal conditions and suppresses the interaction between RIPK3 and MLKL in necroptotic stimulation [27]. Recently, the addition of O-linked β-N-acetylglucosamine on T467 of RIPK3 inhibits RIPK1/RIPK3 necrosome formation [28]. All these necroptosis regulatory mechanisms involve the modulation of necrosome complex formation itself. However, the regulatory mechanism of the post-necrosome step is yet to be elucidated.

Beclin 1 is a core protein of the autophagy process, containing protein-binding domains such as BCL2 homology (BH)-3 domain, coiled-coil domain (CCD), and an evolutionarily conserved domain (ECD) [29, 30]. PIK3C3/Vps34 and Vps15 form a complex with Beclin 1 during autophagy, which subsequently generates PtdIns-3P (phosphatidylinositol 3-phosphate), thus initiating phagophore nucleation [30]. While Beclin 1 is a key component of the autophagic process, it is also involved in various other pathways, including the cell death process. Under apoptotic conditions, active caspases cleave Beclin 1 to N- and C-terminal fragments, thus abrogating its autophagic function [31, 32]. Moreover, the fragmented N-terminus of Beclin 1 moves to the mitochondria membrane to accelerate mitochondria membrane permeabilization and apoptosis [31, 32]. Recently, Song et al. demonstrated the autophagy-independent role of Beclin 1 in ferroptosis [33]. AMP-activated protein kinase-mediated phosphorylation of Beclin 1 induces interaction between Beclin 1 and SLC7A11, which suppresses the function of system X_c_^−^, thereby inhibiting ferroptosis [33].

Here, we demonstrated that Beclin 1 is integrated into the necrosome complex by interacting with the phosphorylated form of MLKL. Beclin 1 binds to CCD of MLKL in necroptosis process, which prevents MLKL oligomerisation and the perforation of the plasma membrane, thus preventing necroptosis. Physiologically, the conditional knockout of Beclin 1 in bone marrow-derived macrophages (BMDM) elevated cell death with the increase of MLKL oligomerisation compared to the control. Overall, these data suggest that Beclin 1 functions as a negative regulator in the post-necrosome step of necroptosis via the suppression of MLKL oligomerisation.

## Materials and methods

### Mice

*Becn1* conditional knockout mice were obtained from the European Mouse Mutant Cell Repository. LyzMCre mice were obtained from M.-S. Lee (Yonsei University) [34]. *Becn1* conditional knockout mice containing knockout first allele were crossed with actin-flippase transgenic mice to obtain *Becn1*floxed allele. *Becn1*floxed mice were crossed with LyzMCre mice to produce macrophage-specific *Becn1* knockout mice. *Becn1* conditional knockout and LyzMCre mice were genotyped via PCR using following primers: *Becn1* F 5′-TTGTACCGTGATTTAGGGCGTTTGC-3′, R 5′-CTCCCAAGTGCTGGGATTAAAGACG-3′; LyzMCre F 5′-GGTCGATGCAACGAGTGATGAGGT-3′, R 5′-CAGCATTGCTGTCACTTGGTCGTG-3′. The Institutional Animal Care and Use Committees of the Laboratory Animal Research Center at Yonsei University approved all the experiments (IACUC-A-201811-822-01). Mice analyses were not randomized. The investigators were not blinded to allocation during experiments and outcome assessment.

### Cell culture, plasmids, and transfection

HT-29 (human colorectal carcinoma; HTB-38; ATCC, Manassas, VA, USA), TC-1 (mouse lung cancer cell line; CRL-2785; ATCC), and Molm-13 (acute myeloid leukemia; ATCC) cells were maintained in Roswell Park Memorial Institute (RPMI; HyClone, Chicago, IL, USA) in 5% CO_2_ at 37 °C. 293T (human embryonic kidney; CRL-3216; ATCC), L929 (mouse fibrosarcoma; CRL-6364; ATCC), and BMDM (mouse bone marrow-derived macrophage) were maintained in Dulbecco’s modified Eagle’s medium (DMEM; HyClone) in 5% CO_2_ at 37 °C. All media were supplemented with 10% fetal bovine serum (HyClone) and 1% penicillin/streptomycin (Invitrogen, Carlsbad, CA, USA). Hank’s Balanced Salt Solution (HBSS) (Thermo Fisher Scientific, Waltham, MA, USA) was used to induce autophagic conditions by preincubation for 2 h in HT-29, TC-1, and L929 cells. All cells were tested mycoplasma contamination using e-Myco™ plus Mycoplasma PCR Detection Kit (#25237; Intron, Seongnam, Gyeonggi, South Korea).

The RIPK1 and RIPK3 constructs (pcDNA3-FLAG–RIPK1 or RIPK3, pcDNA3-HA-RIPK1 or RIPK3) have been described previously [24]. pcDNA3-FLAG-MLKL mutants (T357E/S358D, 1–178, 179–471, Δ1–84, Δ139–180) were generated using site-directed mutagenesis and PCR, respectively. The Beclin 1 construct was provided by HWL (Yonsei University) and subcloned into the pcDNA3-HA, pcDNA3-FLAG, pMSCVpuro-FLAG, and pMSCVhygro vectors using PCR. The pcDNA3-HA-Beclin 1 mutants (1–140, 141–277, 1–244, 141–450, 245–450, Δ141–244 (ΔCCD)) and the pMSCVpuro-FLAG-Beclin 1 mutant (Δ141–244 (ΔCCD)) were generated using PCR. pMSCVpuro-FLAG-Beclin 1 WT, ΔCCD, and pMSCVhygro-FLAG-Beclin 1 resistant to *BECN1* shRNA or siRNA were generated using site-directed mutagenesis with the following primers: F 5′-TCAGAGATACCGTCTAGTTCCTTACGGA-3′, R 5′-TCCGTAAGGAACTAGACGGTATCTCTGA-3′. We obtained and engineered pMSCVpuro-FLAG and pMSCVhygro-FLAG vectors from Addgene (#53178, #75083).

pLKO.1 puro-shBECN1 and pLKO.1 hygro-shRIPK3 were generated using oligo annealing and cloning into an empty vector using following primers: shBECN1 #5F 5′-CCGGGATACCGACTTGTTCCTTACGCTCGAGCGTAAGGAACAAGTCGGTATCTTTTTG-3’, R 5′-AATTCAAAAAGATACCGACTTGTTCCTTACGCTCGAGCGTAAGGAACAAGTCGGTATC-3′, shBECN1 #7F 5′-CCGGCTAAGGAGCTGCCGTTATACTCTCGAGAGTATAACGGCAGCTCCTTAGTTTTTG-3′, R 5′-AATTCAAAAACTAAGGAGCTGCCGTTATACTCTCGAGAGTATAACGGCAGCTCCTTAG-3′, shRIPK3 F 5′-CCGGAACCAGCACTCTCGTAATGATCTCGAGATCATTACGAGAGTGCTGGTTTTTTTG-3′, R 5′-AATTCAAAAAAACCAGCACTCTCGTAATGATCTCGAGATCATTACGAGAGTGCTGGTT-3′. shMLKL (TRCN0000196317) was purchased from Sigma-Aldrich.

For transfection, the plasmids were incubated with polyethyleneimine (PEI) (Sigma-Aldrich) in serum-free media for 20 min, and then added to 293T cells. After incubating for 24 h, the cells were harvested and analysed.

### Generation and validation of *BECN1* knockout and knockdown cell lines

LentiCRISPRv2 vector was obtained from Addgene (Addgene plasmid #52961; Cambridge, MA, USA). As suggested by the CRISPOR online program (http://crispor.tefor.net), single-guide RNA-targeting exon 2 or 3 of the human *BECN1* gene (KO#1; 5′-CACCGCCTGGACCGTGTCACCATCC-3′ or KO#2 and #3; 5′-CACCGCAGGAGGAAGAGACTAACTC-3′) was cloned into lentiCRISPRv2 vector using GeCKO’s cloning protocol. 293T cells were transfected with lentiCRISPRv2-sgBECN1 vectors using packaging plasmids for the production of lentivirus. HT-29 cells were then infected with the lentivirus and selected using 1 μg/mL puromycin treatment for 7 days. After puromycin selection, single-colony selection was performed in order to identify Beclin 1 complete knockout cells, verified by immunoblotting.

To establish a cell line expressing shRNA against Beclin 1, RIPK3, and MLKL, 293T cells were transfected with pLKO.1 puro-shGFP, pLKO.1 puro-shBECN1#5, pLKO.1 puro-shBECN1#7, pLKO.1 puro-shMLKL, and pLKO.1 hygro-shRIPK3 using packaging plasmids to produce a lentivirus. HT-29 and Molm-13 cells were infected with the lentivirus containing shGFP, shBECN1#5, #7, and shRIPK3 and stably transfected cells were selected by treatment with 1 μg/mL puromycin or 400 μg/mL hygromycin for 7 days. To establish a cell line overexpressing shRNA-resistant Beclin 1, 293T cells were transfected with pMSCVpuro-FLAG-Beclin 1 or pMSCVhygro-FLAG-Beclin 1 using packaging plasmids for the production of a retrovirus. HT-29 cells were infected with the retrovirus and stably transfected cells were selected by treatment with 1 μg/mL puromycin or 400 μg/mL hygromycin for 7 days.

### siRNA and transfection

The ON-TARGET plus SMARTpool siRNAs for human BECN1 pool (L-010552-00), BECN1 #5 (J-010552-05), BECN1 #7 (J-010552-07), ATG7 pool (L-020112-00), VPS34 pool (L-005250-00) and mouse Becn1 pool (L-055895-00), Becn1 #7 (J-055895-07), Becn1 #8 (J-055895-08), Atg7 pool (L-049953-00), Vps34 pool (L-063416-00) were purchased from Dharmacon (Dharmacon, Lafayette, CO, USA). Non-targeting pool (siNT, D-001810-10, Dharmacon) was used as a control.

TC-1 and L929 cells were transfected with 10 or 20 nM siRNAs using Lipofectamine RNAiMax (Invitrogen) for 48 h. Dharmafect 1 (Dharmacon) was used for HT-29 cell transfection. siRNA transfection was performed according to the manufacturer’s instructions.

### Chemicals and cell death stimulation

BV6 (S7597), Birinapant (S7015), and Z-VAD-FMK (S7023) were purchased from Selleck Chemicals (Houston, TX, USA). Human TNFα (210-TA-020; R&D Systems, Minneapolis, MN, USA), mouse TNFα (14-8321-62; eBioscience, San Diego, CA, USA), emricasan (254750-02-2; Cayman Chemical, Ann Arbor, MI, USA), GSK’963 (AOB9775; Aobious, Gloucester, MA, USA), GSK’872 (530389, Calbiochem, San Diego, CA, USA), necrostatin-1 (BML-AP309; Enzo Life Sciences, New York, NY, USA), and necrosulphonamide (480073; Calbiochem, San Diego, CA, USA), and anti-TNFα antibody (MAB610, R&D Systems) were purchased from the indicated companies. Necroptotic cell deaths were induced via treatment with following combinations of chemicals: HT-29 hTNFα (30 ng/mL), BV6 (1 µM) or birinapant (1 µM), and Z-VAD-FMK (30 µM); L929 mTNFα (5 ng/mL) and Z-VAD-FMK (10 µM); TC-1 mTNFα (10 ng/mL), BV6 (1 µM), and Z-VAD-FMK (20 µM); primary BMDM mTNFα (20 ng/mL), BV6 (1 µM), and Z-VAD-FMK (20 µM); Molm-13 birinapant (0.05 µM), and Z-VAD-FMK (20 µM) or emricasan (1 µM).

### Cell viability analysis and flow cytometry

To determine cell viability, treated cells were incubated with Cell Titer-Glo for 20 min at room temperature (RT), before being analysed using a luminometer (Cell Titer-Glo Luminescent Cell Viability Assay kit, G7571, Promega, Madison, WI, USA) according to the manufacturer’s instructions. Cell loss was calculated using the following formula: cell loss = 100 – cell viability. For Annexin V and 7-AAD double staining, treated cells were harvested and washed with PBS, and then followed by incubation with Annexin V-FITC (556547; BD Biosciences) and 7-AAD (00-6993-50, eBioscience)in Annexin V binding buffer (51-66121E, BD Biosciences, Franklin Lakes, NJ, USA) for 15 min. For propidium iodide (PI) single staining, treated cells were harvested and washed with PBS, followed by incubation with PI (P4170; Sigma-Aldrich, St. Louis, MO, USA) for 15 min. Dead cells were determined as the PI-positive population. Stained cells were analysed using flow cytometry (BD accuri C6, BD Biosciences). Data were analysed using BD ACCURI C6 PLUS software (BD Biosciences). All cell viability analyses were biologically triplicated and presented as mean ± standard deviation.

### Immunofluorescence microscopy

For immunofluorescence staining, HT-29 cells were cultured in 12-well plates with coverslips. After treatment with necroptosis stimuli for the indicated times, the cells were fixed with 4% paraformaldehyde for 30 min at RT. Anti-p-MLKL (S358) antibody (ab187091; Abcam, Cambridge, United Kingdom) (1:50) and anti-Beclin 1 antibody (sc-48341; Santa Cruz Biotechnology, Dallas, TX, USA) (1:25) were added to each sample in immunofluorescence blocking buffer (PBS with 3% BSA, 1% saponin, and 1% Triton X-100), and then incubated at 4 °C overnight. Each sample was washed with PBS three times. Alexa Fluor 488 anti-mice (A-11029; Thermo Fisher Scientific) and Alexa Fluor 594 anti-rabbit IgG conjugated antibodies (A-11032; Thermo Fisher Scientific) were added to each sample in immunofluorescence blocking buffer (PBS with 3% BSA, 1% saponin, and 1% Triton X-100), and then incubated at RT for 1 h. Nucleus was stained with DAPI for 5 min and analysed by LSM 700 (Carl Zeiss, Oberkochen, Germany).

### Immunoprecipitation and immunoblotting

Cells were lysed with lysis buffer containing 50 mM Tris-HCl (pH 7.5), 150 mM NaCl, 0.5% Triton X-100, 1 mM EDTA, and a protease inhibitor cocktail. Cell lysates were immunoprecipitated by incubating with the indicated antibodies for 2 h, followed by incubation with protein G Sepharose (GE Healthcare, Chicago, IL, USA) for 2 h. Next, samples were eluted in sample buffer and boiled for 5 min. For immunoprecipitation, anti-Beclin 1 (sc-48341; Santa Cruz Biotechnology), caspase-8 (sc-6136; Santa Cruz Biotechnology), FADD (sc-6036; Santa Cruz Biotechnology), HA (sc-7392; Santa Cruz Biotechnology), and FLAG (F3165; Sigma-Aldrich) antibodies were used. Normal mouse IgG (sc-2025) and goat IgG (sc2028) purchased from Santa Cruz Biotechnology were used as controls.

For necrosome complex formation analysis, cells were treated with TNFα and BV6 or birinapant in the presence of Z-VAD-FMK for the indicated times. Cells were lysed in death inducing signalling complex immunoprecipitation buffer containing 50 mM Tris-HCl (pH 7.5), 150 mM NaCl, 1% Triton X-100, 10% glycerol, and 1 mM EDTA with a proteasome inhibitor cocktail. Complexes were purified by immunoprecipitation using anti-caspase-8 (sc-6136; Santa Cruz Biotechnology) and FADD (sc-6036; Santa Cruz Biotechnology) antibodies.

The following antibodies were used for immunoblotting: Beclin 1 (1:1000; sc-11427; Santa Cruz Biotechnology and 1:2000; 3738S; Cell Signaling Technology), ATG7 (1:500; #2631; Cell Signaling Technology, Danvers, MA, USA), human caspase-8 (1:1000; #9746, Cell Signaling Technology), mouse caspase-8 (1:1000; ALX-804-447; Enzo life Science), hFADD (1:500; 610400; BD Bioscience), mFADD (1:1000; 05-486; Merck Millipore, Burlington, MA, USA), RIPK1 (1:1000; #610459; BD Bioscience), hRIPK3 (1:2000; 13526; Cell Signaling Technology), mRIPK3 (1:2000; NBP1-77299; Novus Biologicals, Centennial, Co, USA), p-hRIPK3 (S227) (1:2000; ab209384; Abcam), hMLKL (1:2000; GTX107538; GeneTex, Irvine, CA, USA), mMLKL (1:2000; AP14272b; Abgent, San Diego, CA, USA), p-hMLKL (S358) (1:2000; ab187091; Abcam), p-mMLKL (S345) (1:2000; ab196436; Abcam), VPS34 (1:1000; #4263; Cell Signaling Technology), Rab5 (1:500; sc-46692; Santa Cruz Biotechnology), IκBα (1:1000; #4814; Cell Signaling Technology), HA (1:5000; 11867431001; Roche, Basel, Switzerland), FLAG (1:3000; F3165, F7425; Sigma-Aldrich), and β-actin (1:5000; A5316; Sigma-Aldrich). The antibodies were purchased from the indicated companies.

### Gel filtration chromatography analysis

HT-29 cells stably expressing indicated shRNAs treated with hTNFα (30 ng/mL), BV6 (1 µM), and Z-VAD-FMK (30 µM) for the indicated times. Cells were harvested in seven 100 mm culture dishes and lysed with 2.5 mL of lysis buffer containing 50 mM Tris-HCl (pH 7.5), 150 mM NaCl, 0.5% Triton X-100, and 1 mM EDTA. The lysates were injected into AKTA Prime Plus and separated using a HiLoad 16/600 Superdex 200 column at a flow rate of 0.4 ml/min (GE Healthcare). Fractions of 2 mL were collected from 42 to 102 mL, and samples were analysed by western blotting using the indicated antibodies. Molecular weight markers were determined using gel filtration calibration kits (GE Healthcare).

### Triton X-114 phase partitioning

Triton X-114 phase partitioning was performed using the multiple washing method. Briefly, cells were resuspended in aqueous 2% (w/v) Triton X-114 containing 10 mM Tris-HCl (pH 7.5) and 150 mM NaCl. The suspension was incubated on ice for 30 min with frequent vortexing, and then centrifuged at 10,000 *g* at 4 °C for 20 min to sediment the pellet fraction (PF). The supernatant was collected and incubated for 15 min at 37 °C to achieve phase partitioning. The mixture was centrifuged at 5000 *g* at 25°C for 30 min, and the upper aqueous phase (AP) and lower detergent phase (DP) were carefully collected. The AP was further purified by adjusting the Triton X-114 concentration to 2% (w/v) and repeating the phase partitioning, as above. Similarly, the DP was further purified by adding an equal volume of aqueous 0.06% (w/v) Triton X-114 and repeating the phase partitioning process.

### MLKL oligomerisation

Non-reducing samples were prepared by lysing cells with sample buffer containing 100 nM Tris-HCl, 4% SDS, 20% glycerol, 20 mM EDTA, and bromophenol blue without β-mercaptoethanol. For reducing samples, cells were lysed with sample buffer containing 100 nM Tris-HCl, 4% SDS, 20% glycerol, 20 mM EDTA, bromophenol blue, and 200 mM β-mercaptoethanol. Both non-reducing and reducing samples were boiled for 10 min, and then analysed via SDS-PAGE.

### Preparation of L929 conditioned medium

L929 cells were plated 5 × 10^5^ cells in 150 T flask containing 50 mL of 10% fetal bovine serum-supplemented DMEM medium, and then incubated in 5% CO_2_ at 37 °C for one week. The supernatant was collected and filtered through 0.45 µm filter and stored at −70 °C.

### Preparation of murine bone marrow-derived macrophages (BMDM)

Bone marrow cells were obtained from 9-week-old genotyped C57BL/6 mice. After euthanasia using CO_2_, the hind legs were extracted by surgery. The femur was separated from the tibia by cutting at the knee joint, and the bone marrow cells in the hind legs were extracted by flushing using a syringe containing serum-free media several times into DMEM medium containing 10% fetal bovine serum. Extracted cells were filtered through a 40 μm nylon cell strainer (SPL, Pocheon, Gyeonggi, South Korea) to remove any debris. The extracted cells were then collected via centrifugation. The supernatants were discarded and the pellets were resuspended in media before being transferred into a non-coating cell culture dish. To remove fibroblast contamination, cells were incubated in 5% CO_2_ at 37 °C for 4 h. Cells floating in the supernatant were transferred into new non-coating culture dishes, and then added L929 conditioned medium up to 10% for one week. Differentiated BMDM cells were sub-cultured for further analysis.

### Xenograft study

In total, 5 × 10^5^ Molm-13 cells were prepared for each mouse and resuspended in 50 μL of PBS. Cells were mixed with 50 μL of Matrigel matrix (354234, BD Sciences). The mixtures of cells and matrigels were subcutaneously injected in the flank of 6-week-old female Balb/c nude mice (Narabiotech, Seoul, Korea). After one week, mice with xenograft tumours were inoculated with vehicle or birinapant (2 mg/kg) plus emricasan (1 mg/kg) by i.p. injection in every two days. Tumour growth was measured as described previously [35]. After 18 days from tumour inoculation, mice were sacrificed, and tumour volumes and masses were determined as described previously [35]. We analysed six mice for each group. Animal experiments were approved by the Institutional Animal Care and Use Committee of the Laboratory Animal Research at Yonsei University (IACUC-A-201905-916-01). Xenograft analyses were not randomized. The investigators were not blinded to allocation during experiments and outcome assessments.

### Statistical analysis

Statistical significance was assessed using Student’s *t* test in GraphPad Prism software (Ver. 5.01; La Jolla, CA, USA).

## Results

### Beclin 1 protects cells from necroptosis in an autophagy-independent manner

**Fig. 1:**
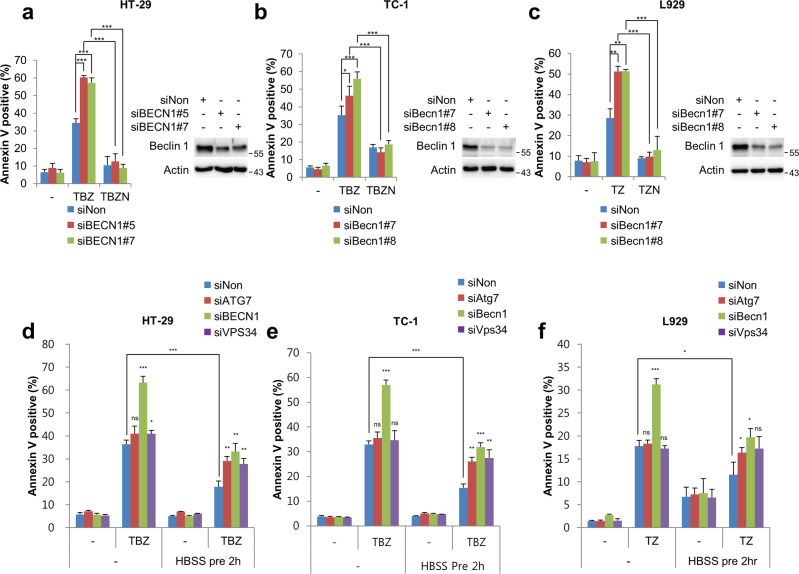
Beclin 1-depleted cells are sensitised to necroptosis in an autophagy-independent manner. **a** HT-29 cells depleted of Beclin 1 by the indicated siRNAs were treated with 30 ng/mL TNFα, 1 μM BV6, and 30 μM Z-VAD-FMK (TBZ) in the presence or absence of 45 μM Nec-1 for 4 h. **b** TC-1 cells depleted of Beclin 1 by indicated siRNAs were treated with 10 ng/mL TNFα, 1 μM BV6, and 20 μM Z-VAD-FMK (TBZ) in the presence or absence of Nec-1 for 3 h. **c** L929 cells depleted of Beclin 1 by the indicated siRNAs were treated with 5 ng/mL TNFα and 10 μM Z-VAD-FMK (TZ) in the presence or absence of Nec-1 for 3 h. **a**–**c** Knockdown efficiencies were determined by western blotting. After inducing necroptosis, cells were stained with annexin V-FITC and 7-AAD, before analysing by flow cytometry. **d** HT-29, **e** TC-1, and **f** L929 cells depleted of autophagy factors by the indicated siRNAs were treated with TBZ or TZ under either normal conditions or autophagic conditions induced by preincubation with HBSS media. After treatment for inducing necroptosis, cells were stained with annexin V-FITC and 7-AAD, before being analysed by flow cytometry. Data are the mean ± standard deviation (S.D.), *n* = 3, with ns non-significance, *P < 0.05, **P < 0.01, and ***P < 0.001 at each point compared to indicated graph with the two-sided Student’s *t* test (**a**–**f**).

Beclin 1 is a modulator of the apoptosis pathway [31]. To further identify whether Beclin 1 is involved in TNFα-induced necroptosis pathway, HT-29, TC-1, and L929 cells were transfected with Beclin 1 siRNAs followed by treatment with TNFα, Smac mimetic (BV6), and Z-VAD-FMK, a pan-caspase inhibitor (TBZ). Interestingly, Beclin 1 depletion sensitised all three cell lines to TNFα-induced necroptosis (Fig. 1a–c and Supplementary Fig. 1a–c). However, treatment with necrostatin-1, a RIPK1 kinase inhibitor, blocked the necroptotic cell death caused by TBZ even in the ablated state of Beclin 1, suggesting that Beclin 1 has a protective role in RIPK1-dependent necroptosis (Fig. 1a–c and Supplementary Fig. 1a–c). Since Beclin 1 is a main component of the autophagy process, we further confirmed the effect of autophagy in necroptosis regulation. As previously reported, cells starved with HBSS incubation displayed resistance to TNFα-induced necroptosis (Fig. 1d–f and Supplementary Fig. 1d–f) [36]. The depletion of ATG7, VPS34, and Beclin 1, which are key components of autophagy, reversed the inhibitory effect of HBSS incubation in TNFα-induced necroptosis, indicating that autophagy might exhibit a negative function on necroptosis (Fig. 1d–f and Supplementary Fig. 1d–f). It is worth noting that ATG7 and VPS34 depletions did not affect TNFα-induced necroptosis under normal conditions, whereas Beclin 1 depletion promoted TNFα-induced necroptosis in normal as well as HBSS medium (Fig. 1d–f and Supplementary Fig. 1d–f). Collectively, these results indicate that Beclin 1 is involved in necroptosis in an autophagy-independent manner.

### Beclin 1 incorporated into the necrosome complex in an MLKL-dependent manner suppresses MLKL oligomerisation

**Fig. 2:**
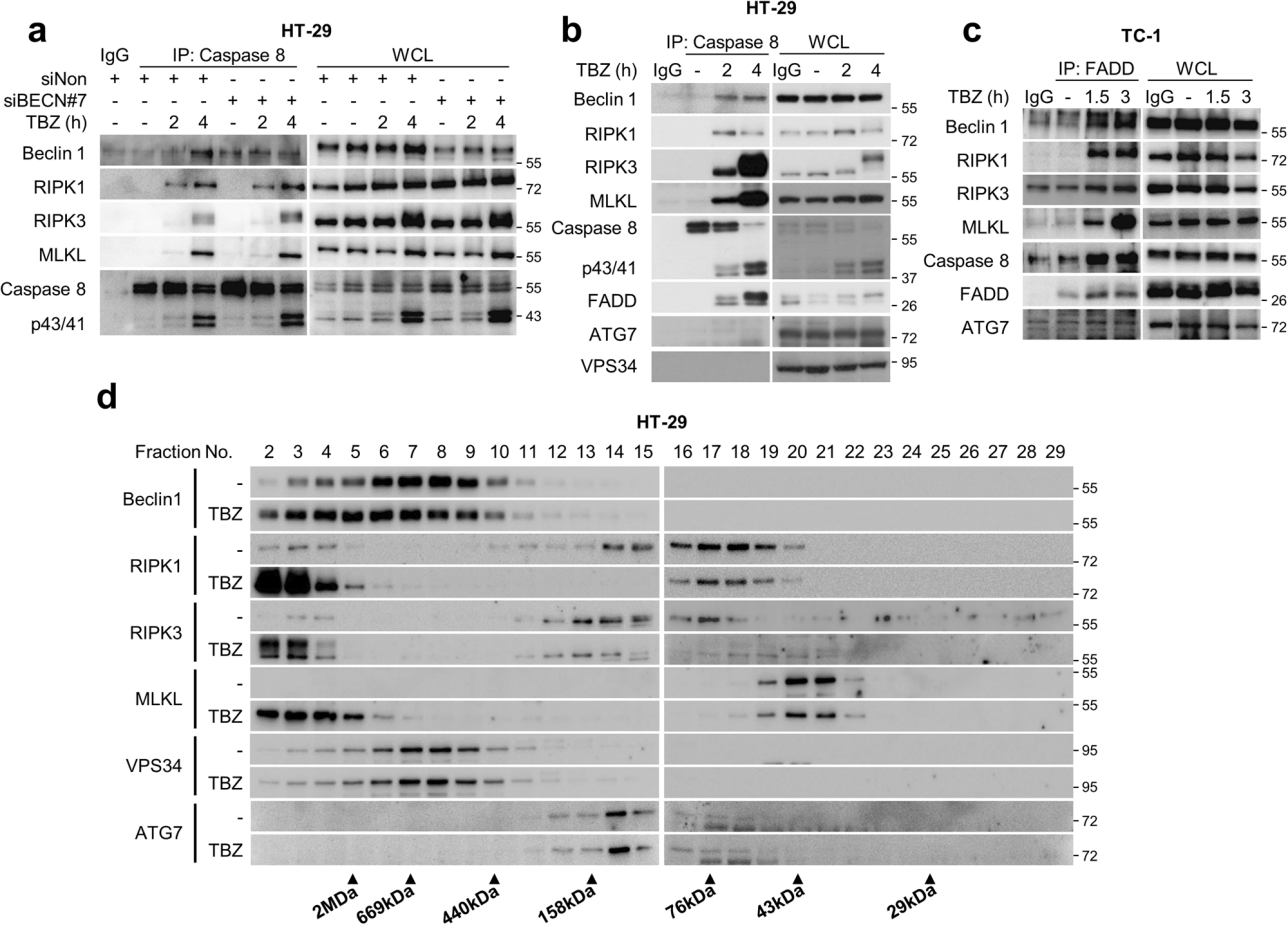
Beclin 1 is incorporated into the necrosome complex. **a** HT-29 cells were transfected with the indicated siRNAs for 48 h, and then treated with TBZ for the indicated times. After treatment, the cells were lysed with lysis buffer and incubated with the anti-caspase-8 antibody. Samples were precipitated by incubating with protein G agarose, followed by immunoblotting analysis using the indicated antibodies. HT-29 (**b**) and TC-1 cells (**c**) were treated with TBZ for the indicated times. After treatment, the cells were lysed with lysis buffer and then immunoprecipitated using the anti-caspase-8 antibody (**b**) or anti-FADD antibody (**c**). The protein levels were determined by immunoblotting using the indicated antibodies. **d** Control and TBZ treated-HT-29 cells were lysed using lysis buffer and then fractionated according to their molecular size by gel filtration chromatography. The samples were analysed by immunoblotting.

**Fig. 3:**
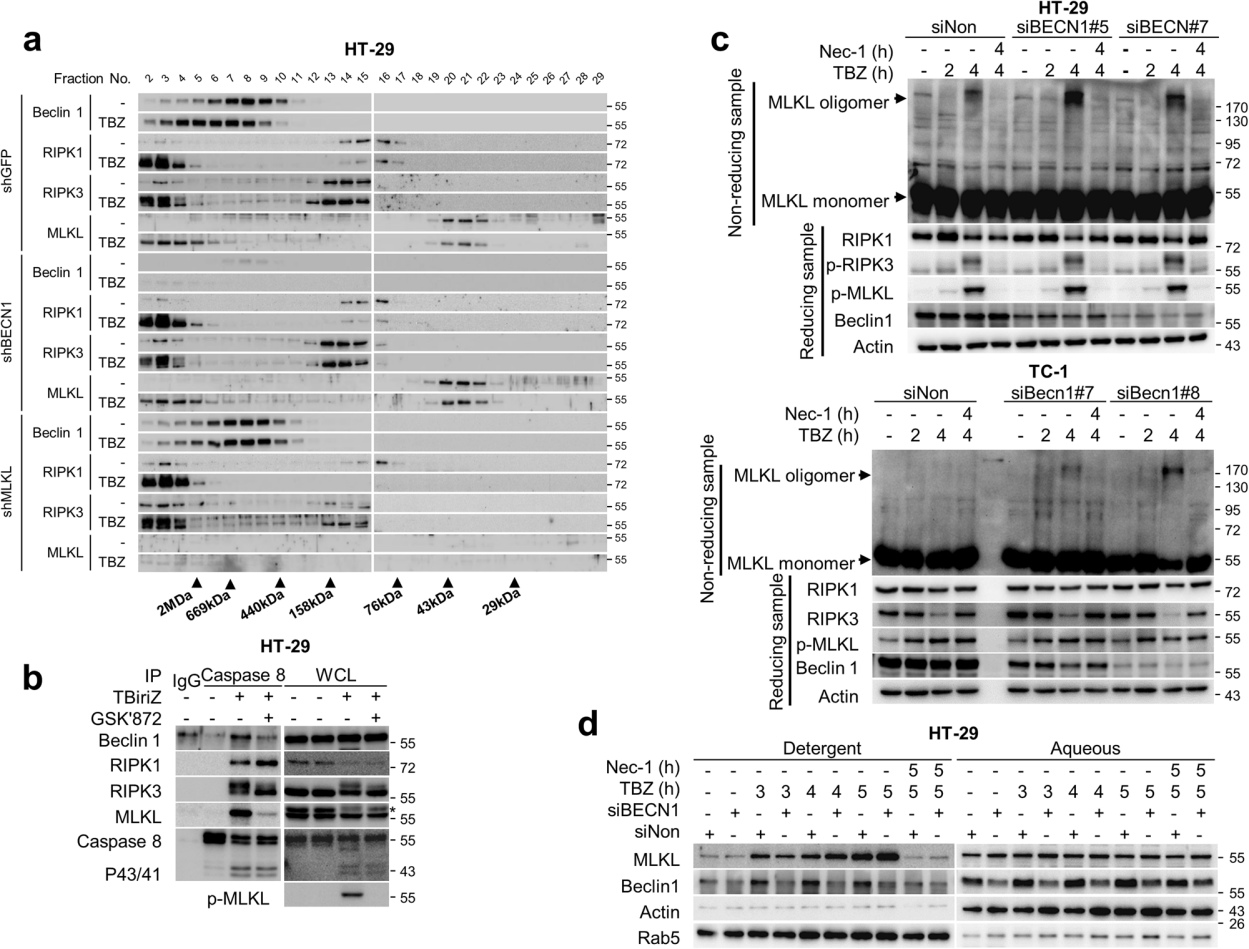
Beclin 1 inhibits MLKL oligomerisation in the necrosome complex. **a** HT-29 cells expressing shGFP, shBECN1, and shMLKL were treated with TBZ. After treatment, the cells were lysed using lysis buffer and then fractionated according to their molecular size by gel filtration chromatography. The samples were analysed by immunoblotting. **b** HT-29 cells were treated with 30 ng/mL TNFα, 1 μM Birinapant, and 30 μM Z-VAD-FMK (TBiriZ) in the presence or absence of 2 μM GSK'872 for 5 h. After treatment, the cell lysates were immunoprecipitated using anti-caspase-8 antibody and then analysed by immunoblotting. **c** HT-29 and TC-1 cells were transfected with the indicated siRNAs, and then treated with TBZ in the presence or absence of Nec-1 for the indicated times. After treatment, the cell lysates were lysed under non-reducing conditions to detect MLKL oligomerisation or under reducing conditions and then analysed by immunoblotting. **d** HT-29 cells were transfected with the indicated siRNAs for 48 h and then treated with TBZ in the presence or absence of Nec-1 for the indicated time points. After treatment, the cell lysates were lysed using Triton X-114 buffer to divide them into detergent and aqueous phases. Each lysate was analysed by immunoblotting.

Maintaining the levels of proteins involved in necroptosis is necessary for modulating necroptosis. However, under Beclin 1-depleted conditions, no obvious changes were observed in the protein levels of necroptotic factors in HT-29, TC-1, or L929 cells (Supplementary Fig. 2a). There was also no change in IκBα degradation caused by TNFα treatment under Beclin 1 ablation (Supplementary Fig. 2b). Moreover, Beclin 1 ablation had no effect on the formation of the RIPK1/RIPK3/MLKL necrosome complex (Fig. 2a). To determine whether autophagy factors are incorporated in the complex, the presence of autophagy factors in the necrosome complex was investigated. The results showed that in HT-29 and TC-1 cells, only Beclin 1, and not ATG7 and VPS34, was recruited to the necrosome complex in response to TBZ (Fig. 2a–c). Notably, the incorporation of Beclin 1 in the complex occurred at 2 and 1.5 h in HT-29 and TC-1 cells, respectively, when the complete RIPK1/RIPK3/MLKL complex was formed, suggesting that Beclin 1 could be involved in the later stages of the necroptotic process (Fig. 2b, c). We have further explored the integration of Beclin 1 into the necrosome complex through gel filtration analysis using HT-29 cells. As previously reported, the necroptotic stimulus by TBZ triggered the translocation of RIPK1, RIPK3, and MLKL into a large multi-protein complex (Fig. 2d). While VPS34 and ATG7 did not translocate to the macromolecular fractions in response to TBZ, Beclin 1 was translocated upon treatment with TBZ (Fig. 2d). The next question was whether Beclin 1 could affect the macromolecular translocation of necroptosis factors under necroptosis stimulation. When Beclin 1 was depleted, there was no noticeable change in the translocation of RIPK1, RIPK3, and MLKL to their macromolecular fractions upon TBZ treatment (Fig. 3a). Interestingly, when MLKL was depleted using MLKL-specific shRNAs, Beclin 1 translocation into high molecular weight fractions was strongly reduced (Fig. 3a). These observations were further supported when GSK’872, an inhibitor of RIPK3, was used upon the cells with necroptotic stimuli, which resulted in a significant decrease of MLKL and Beclin 1 binding to the necrosome complex, suggesting that the incorporation of phosphorylated MLKL into the necrosome complex may be required for the recruitment of Beclin 1 into the complexes (Fig. 3b).

Beclin 1 had no clear effect on the levels of necrosome complex formation and required MLKL to be incorporated into the complex. Hence, we investigated the effect of Beclin 1 on the status of MLKL phosphorylation or oligomerisation. Beclin 1 depletion promoted MLKL oligomerisation without affecting MLKL phosphorylation in HT-29 and TC-1 cells (Fig. 3c). It is well demonstrated previously that membrane localisation of MLKL follows MLKL oligomerisation [8, 9, 11–13, 37]. When the membrane localisation of MLKL was analysed, there was an increased of MLKL under Beclin 1-depleted conditions in HT-29 cells with necroptotic stimulus for more than 4 h (Fig. 3d). Overall, these results suggest that Beclin 1 is recruited into the necrosome complex in an MLKL-dependent manner and subsequently restricts necroptosis via the suppression of MLKL oligomerisation, which retards translocalisation into the plasma membrane.

### Beclin 1 is incorporated into the necrosome complex via the interaction with phosphorylated MLKL

**Fig. 4:**
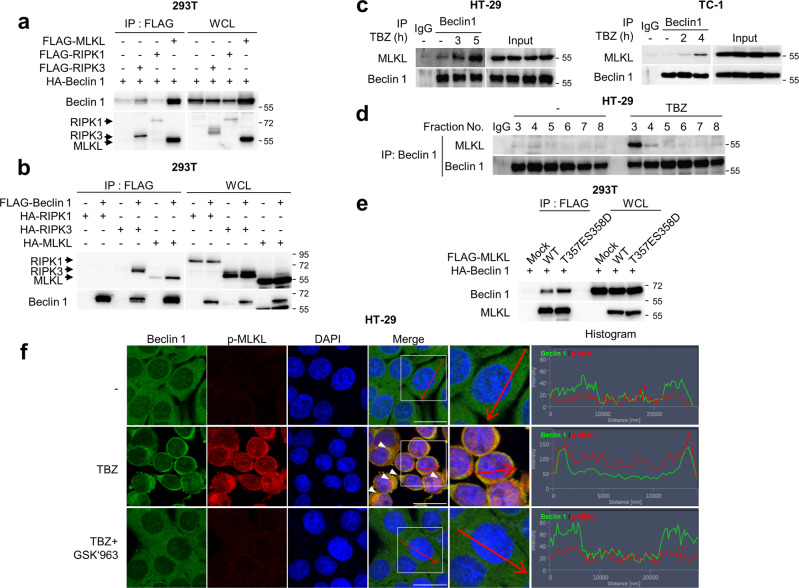
Phosphorylation of MLKL is required for the recruitment of Beclin 1 into the necrosome complex. **a**, **b** 293T cells were transfected with the indicated plasmids and then immunoprecipitated using anti-FLAG antibody. The samples were analysed by immunoblotting using anti-FLAG and HA antibodies. **c** HT-29 and TC-1 cells were treated with TBZ for the indicated times. After treatment, the cell lysates were immunoprecipitated using anti-Beclin 1 antibody and then analysed by immunoblotting. **d** HT-29 cells were treated with TBZ and fractionated according to their molecular size. After fractionation of the cell lysates, each fraction was immunoprecipitated using anti-Beclin 1 antibody, followed by immunoblot analysis. **e** 293T cells were transfected with the indicated plasmids. The cells were lysed using a lysis buffer and then immunoprecipitated using anti-FLAG antibody. The samples were analysed by immunoblotting. **f** HT-29 cells were treated with TBZ in the presence or absence of 100 nM GSK’963 for 5 h. After treatment, the cells were fixed and stained using anti-Beclin 1 and p-MLKL antibodies, and DAPI. The histogram represents the subcellular integrity of Beclin 1 and p-MLKL in the areas indicated by the red arrows. The boxed areas are shown at higher magnification on the right. Scale bars = 20 μm.

To find which necroptosis-related proteins interact with Beclin 1, immunoprecipitation analyses were performed (Fig. 4a, b). While the interaction between Beclin 1 and RIPK3 is interesting, Beclin 1 was incorporated into the necrosome complex in an MLKL-dependent manner. Hence, we decided to investigate the interaction between Beclin 1 and MLKL rather than RIPK3. As expected, the interaction between endogenous Beclin 1 and MLKL was enhanced upon necroptotic stimulus in HT-29 and TC-1 cells (Fig. 4c). Furthermore, when the interaction between Beclin 1 and MLKL was analysed in the gel filtration fractions from Fig. 2d using immunoprecipitation by Beclin 1 antibody, we only observed the interactions between the two proteins in the fractions treated with TBZ but not in the control fractions, indicating that Beclin 1 forms a complex with MLKL in response to necroptotic stimulus (Fig. 4d).

The phosphorylation of human MLKL at threonine 357 and serine 358 by RIPK3 leads to the conformational change of MLKL from the CCD masked form to the CCD unmasked form, which enables MLKL oligomerisation (Fig. 7e) [38–40]. Enhancement of the interaction between Beclin 1 and MLKL upon TBZ treatment led us to speculate whether the phosphorylation of MLKL is required for strong binding between the two proteins. As shown in Fig. 4e, the phosphomimetic mutant of MLKL bound to Beclin 1 more strongly than the MLKL wild type (Fig. 4e). The interaction between the endogenous phosphorylated MLKL and Beclin 1 was further confirmed by confocal analysis. Interestingly, Beclin 1 moved to a specific locus in the cytoplasm in response to the necroptotic stimulus of TBZ, simultaneously co-localising with phosphorylated MLKL as shown in the merged figure (Fig. 4f, arrowheads). In particular, when the areas in which MLKL was aggregated were treated with TBZ, they showed co-localisation of MLKL and Beclin 1 (White arrowheads). The treatment of GSK’963, a RIPK1 inhibitor, with TBZ completely blocked the formation of the phosphorylation of MLKL as detected by the disappearance of the red immunofluorescence and the disperse distribution of the Beclin 1, indicating that the co-localisation of Beclin 1 with MLKL requires the formation of a necrosome complex (Fig. 4f). These observations imply that Beclin 1 might be associated at the site of MLKL complex formation plausibly restricting the perforating complexes of MLKL. Collectively, these results imply that Beclin 1 is recruited into the necrosome complex via its interaction with the phosphorylated and thus CCD unmasked forms of MLKL.

### Ablation of Beclin 1 enhances the pore-formation of oligomerised MLKL

**Fig. 5:**
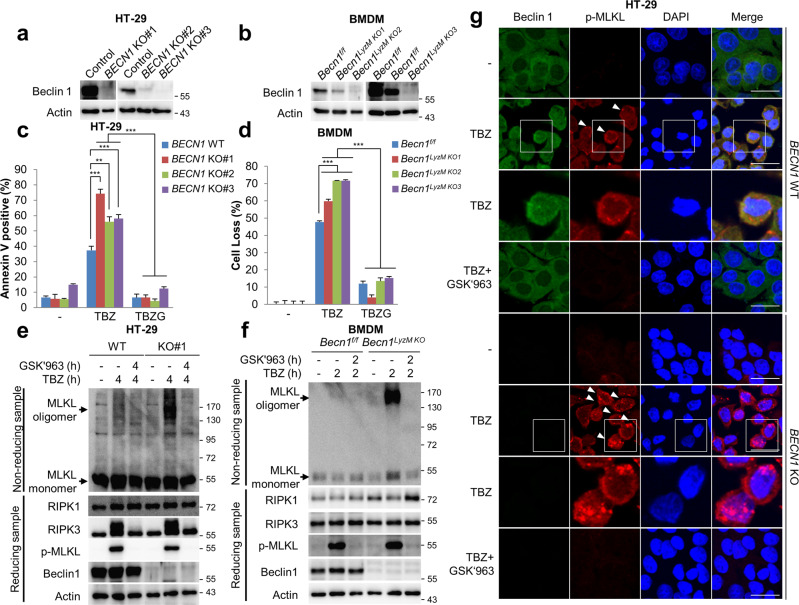
Beclin 1 knockout promotes necroptosis. **a** Beclin 1 protein levels were determined by immunoblotting in Beclin 1 wild type (WT) or knockout (KO) HT-29 cells. **b** Beclin 1 protein was detected by immunoblotting in *Becn1*^*F/F*^ and *Becn1*^*LyzMKO*^ BMDM. **c**, **e** Beclin 1 WT or KO HT-29 cells were treated with TBZ in the presence or absence of GSK'963 (TBZG) for 4 h. After treatment, the cells were stained with annexin V-FITC and 7-AAD for 20 min, and then analysed via flow cytometry (**c**). To analyse MLKL oligomerisation, the cells were lysed under non-reducing conditions for MLKL oligomerisation or under reducing conditions, and then analysed by immunoblotting (**e**). Flow cytometry data are the means ± S.D., *n* = 3, with **P < 0.01 and ***P < 0.001 at each point compared to indicated graph with the two-sided Student’s *t* test (**c**). **d**, **f** *Becn1*^*F/F*^ and *Becn1*^*LyzMKO*^ BMDMs were treated with 20 ng/mL TNFα, 1 μM BV6, and 20 μM Z-VAD-FMK (TBZ) in the presence or absence of 100 nM GSK’963 (TBZG) for 2 h. After treatment, the cell loss was determined via cell titer-glo (**d**). The cell loss data are the means ± S.D., *n* = 3, with ***P < 0.001 at each point compared to indicated graph with the two-sided Student’s *t* test (**d**). To analyse MLKL oligomerisation, the cells were lysed under non-reducing conditions for MLKL oligomerisation or under reducing conditions, and then analysed by immunoblotting (**f**). **g** Beclin 1 WT and KO HT-29 cells were treated with TBZ in the absence or presence of GSK’963 for 5 h. After treatment, the cells were fixed and stained with the anti-Beclin 1, p-MLKL antibodies, and DAPI. The boxed areas are shown at higher magnification to the below. Scale bars = 20 μm.

While we detected the enhancement of oligomerisation of MLKL and necroptotic cell death upon Beclin 1 knockdown, it is still unclear whether the absence of Beclin 1 could promote the formation of phosphorylated MLKL complexes observed in Fig. 4f. To more precisely assess the role of Beclin 1 in this process, we prepared three Beclin 1 knockout HT-29 cell lines using the CRISPR/Cas9 system and three Beclin 1 knockout BMDM from Beclin 1 macrophage-specific knockout mice (Fig. 5a, b and Supplementary Fig. 3). As shown in Fig. 5c, d, all Beclin 1 knockout cell were sensitised to the necroptotic stimulus (Fig. 5c, d and Supplementary Fig. 4a). The increased oligomerisation of MLKL was also observed (Fig. 5e, f and Supplementary Fig. 4b). When HT-29 WT or *BECN1 KO#1* cells were treated with TBZ, we were able to observe the dotted co-localisation of the endogenous Beclin 1 and phosphorylated MLKL (Fig. 5g, *BECN1*, WT, arrowheads). However, there was a great increase in the number and intensities of the dotted complexes of MLKL in the *BECN1 KO#1* cells compared with HT-29 WT cells (Fig. 5g, square). The phosphorylated MLKL was no longer detected under the treatment of GSK’963 indicating the dependency of these processes on necroptosis (Fig. 5g). Next, we wondered whether the increases of cell death and MLKL oligomerisation in Beclin 1 knockout HT-29 cells are solely dependent on the ablation of Beclin 1. We re-expressed wild type Beclin 1 in Beclin 1 knockout HT-29 cells (Supplementary Fig. 5a). The expression of wild type Beclin 1 in Beclin 1 knockout HT-29 cells reduced TBZ-induced cell death as well as MLKL oligomerisation, indicating that the escalated necroptosis in Beclin 1 KO cells could be due to the absence of Beclin 1 and not by other factors (Supplementary Fig. 5b, c). Finally, in order to further analyse the effects of Beclin 1’s ability to suppress the formation of dotted complexes of phosphorylated MLKL, we constructed an HT-29 cell line that was depleted of Beclin 1 using lentivirus expressing *BECN1* shRNA. Subsequently, we overexpressed shRNA-resistant Beclin 1 using retroviral system. Upon knockdown of Beclin 1, we observed the increased necroptotic cell death and dotted complexes of phosphorylated MLKL, while these were reversed by Beclin 1 overexpression (Supplementary Fig. 5d–g). Overall these data indicate that the incorporation of Beclin 1 hampers the formation of the functional complexes of phosphorylated MLKL perforating the plasma membrane and thus suppresses necroptosis.

### Coiled-coil domain of Beclin 1 is responsible for Beclin 1-mediated necroptosis regulation

Since Beclin 1 functions as a negative integral component of necrosome complexes, the domain mapping analyses between Beclin 1 and MLKL were carried out. Wild type and fragments of the CCD of Beclin 1 were capable of interacting with MLKL (Supplementary Fig. 6a, b). The N-terminal fragment of MLKL was also found to be important for binding to Beclin 1 (Supplementary Fig. 6c, d). We next analysed each CCD of MLKL by producing two deletion mutants, Δ1–84 and Δ139-180, which are known to play an essential role in maintaining MLKL structural integrity [9]. Both MLKL deletion mutants bound to Beclin 1, suggesting that the two CCDs of MLKL may redundantly interact with Beclin 1’s CCD (Supplementary Fig. 6e).

**Fig. 6:**
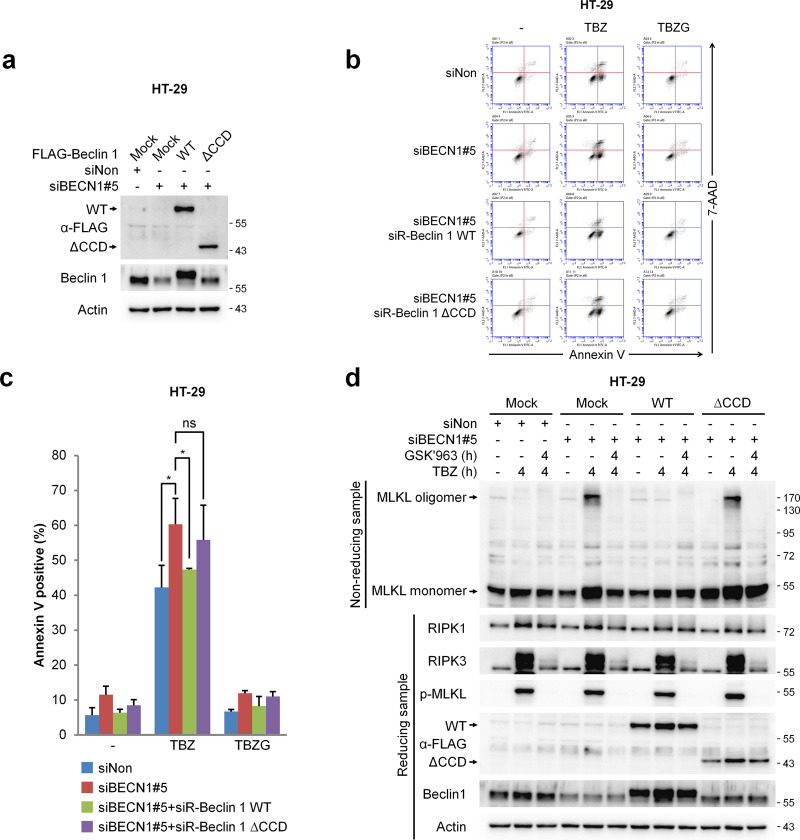
Beclin 1 but not Beclin 1 ΔCCD prevents necroptosis by suppressing MLKL oligomerisation. **a**–**d** HT-29 cells stably expressing FLAG-siBECN1#5-resistant Beclin 1 wild type (WT), or Beclin 1 CCD-truncated mutant (ΔCCD) were transfected with siNon and siBECN1#5 for 48 h. **a** The protein levels were determined by immunoblotting. **b**, **c** To analyse necroptotic population, the cells were treated with TBZ in the presence or absence of GSK'963 (TBZG) for 4 h. After treatment, the cells were stained with annexin V-FITC and 7-AAD, followed by flow cytometry analysis. Data are expressed as the mean ± S.D., *n* = 3, with *P < 0.05 and ns non-significance at each point compared to the indicated graph with the two-sided Student’s *t* test. **d** To analyse MLKL oligomerisation, the cells were treated with TBZ in the presence or absence of GSK'963 (TBZG) for 4 h. After treatment, the cell lysates were lysed under non-reducing conditions for MLKL oligomerisation or under reducing conditions, and were then analysed by immunoblotting.

**Fig. 7:**
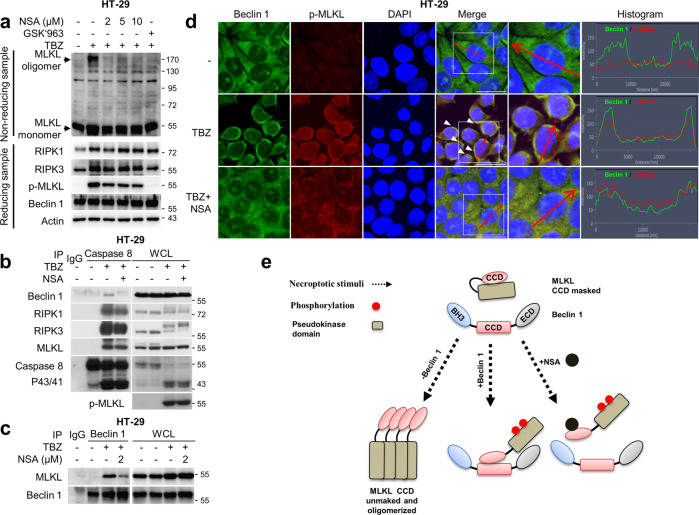
NSA and Beclin 1 compete for CCD of MLKL. **a** HT-29 cells were treated with TBZ in the presence or absence of GSK’963 or NSA. After treatment, cell were lysed under non-reducing conditions for MLKL oligomerisation or under reducing conditions, and then analysed by immunoblotting. **b** HT-29 cells treated with TBZ in the presence or absence of 5 μM NSA for 5 h. After treatment, the cell lysates were immunoprecipitated using the anti-caspase-8 antibody, and then analysed by immunoblotting. **c** HT-29 cells treated with TBZ in the presence or absence of NSA were lysed and immunoprecipitated using the anti-Beclin 1 antibody, followed by immunoblotting analysis. **d** HT-29 cells treated with TBZ in the presence or absence of NSA for 5 h were fixed and stained with the anti-Beclin 1, p-MLKL antibodies, and DAPI. The histogram represents the subcellular integrity of Beclin 1 and p-MLKL of the areas indicated by the red arrows. The boxed areas are shown at higher magnification to the right. Scale bars = 20 μm. **e** Scheme for the hypothetical model of interaction between Beclin 1 and MLKL with necroptotic stimuli in the presence or absence of NSA.

We further tested the importance of CCD of Beclin 1 in preventing MLKL oligomerisation by expressing wild type Beclin 1 or its deletion mutant, ΔCCD, having resistance to Beclin 1 siRNA in HT-29 cells depleted of Beclin 1 siRNA (Fig. 6a). When the cells depleted of Beclin 1 were exposed to necroptotic stimuli, wild type Beclin 1 expression was able to suppress TBZ-induced necroptosis, while ΔCCD expression could not (Fig. 6b, c). Accordingly, the enhanced oligomerisation of MLKL in HT-29 cells depleted of Beclin 1 was also diminished by the expression of the wild type Beclin 1, while ΔCCD failed to repress MLKL oligomerisation (Fig. 6d). To further characterize the nature of the interaction between Beclin 1 and MLKL CCDs, necrosulfonamide (NSA), an MLKL inhibitor, was employed. NSA is known to bind to cys86 in the CCD of MLKL, which abrogates MLKL oligomerisation and thus represses necroptosis [11, 41]. When NSA was employed to treat cells under necroptotic stimuli, we observed that the formation of MLKL oligomerisation was completely blocked, as previously reported (Fig. 7a) [11]. Unexpectedly, treatment of NSA restricted the incorporation of Beclin 1 into the necrosome complex and the interaction between Beclin 1 and MLKL, indicating that NSA might block the incorporation of Beclin 1 into the necrosome complex (Fig. 7b, c). Corroborating these results, NSA was found to suppress the co-localisation of Beclin 1 and phosphorylated MLKL in the cytoplasmic area upon treatment with TBZ (Fig. 7d).

Taken together, these results indicate that the CCD of Beclin 1 is required for the association with CCD of MLKL, which would prevent the oligomerisation of MLKL via its own CCD. Moreover, NSA competes against Beclin 1 for MLKL interaction, suggesting that the CCD of Beclin 1 might interact with the motif of MLKL CCD interacting with NSA (Fig. 7e).

### AML cells are sensitised to necroptosis in vitro and in vivo after Beclin 1 depletion

**Fig. 8:**
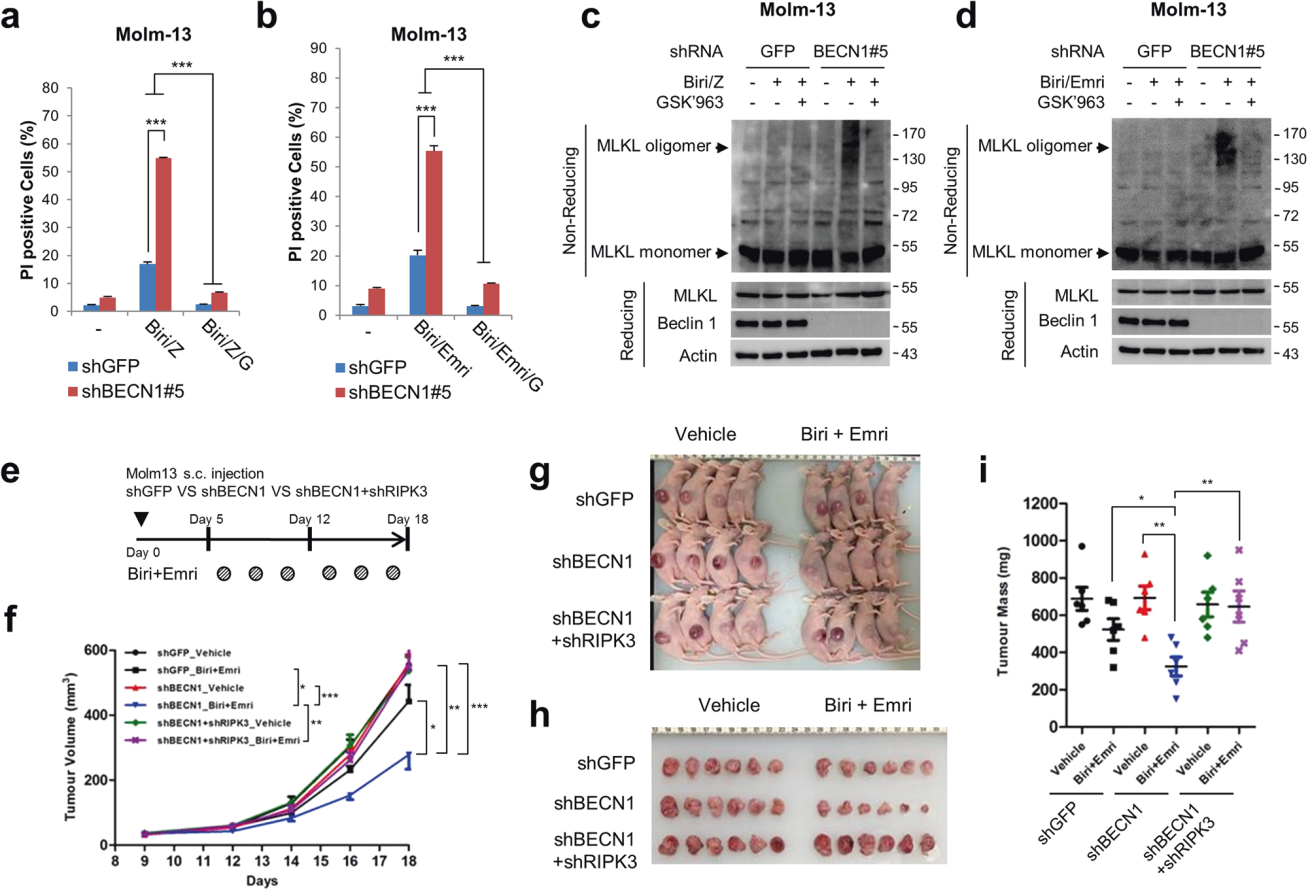
Beclin 1 depletion in Molm-13 promotes necroptosis in vitro and in vivo. Molm-13 cells expressing shGFP and shBECN1 #5 were treated with 0.05 μM birinapant and 20 μM Z-VAD-FMK (Biri/Z) (**a**) or 1 μM emricasan (Biri/Emri) (**b**) in the presence or absence of 100 nM GSK'963 for 10 h, and then stained with propidium iodide (PI) for 15 min. After staining, the cell death was determined by flow cytometry. The data are mean ± S.D., *n* = 3, with ***P < 0.001 at each point compared to the indicated graph using a two-sided Student’s *t* test. Molm-13 cells expressing indicated shRNAs were treated with 0.05 μM birinapant and 20 μM Z-VAD-FMK (Biri/Z) (**c**) or 1 μM emricasan (Biri/Emri) (**d**) in the presence or absence of 100 nM GSK'963 for 10 h. The cells were lysed under reducing conditions or under non-reducing conditions for MLKL oligomerisation, and analysed by immunoblotting. **e** Scheme for the subcutaneous tumour xenograft model. **f**–**i** 5 × 10^5^ Molm-13 cells expressing indicated shRNAs were subcutaneously injected in the flank of 6-week-old female nude mice. After one week, the mice bearing tumours were injected with birinapant (2 mg/kg) plus emricasan (1 mg/kg) as indicated in (**e**) by i.p. injection for 2 weeks. Tumour growth (**f**), tumour-bearing mice (**g**), resected tumours (**h**), tumour masses (**i**) are shown. Data are means and individual data points from *n* = 6 mice, with *P < 0.05, **P < 0.01, ***P < 0.001, and n.s. = non-significance according to the two-tailed Mann–Whitney test.

The role of Beclin 1 as an anti-necroptotic factor prompted us to further explore the effect of Beclin 1 depletion, as an anti-tumour therapy. Molm-13, an acute myeloid leukaemia (AML) cell line, expresses RIPK3 and is known to undergo necroptosis [42–44]. The autocrine TNFα signalling in Molm-13 cells has been shown to be sufficient to induce necroptotic cell death, mediated by Smac mimetic and caspase inhibitor; therefore, we used the combination of Smac mimetic and caspase inhibitor for further necroptosis stimulation in Molm-13 cells (Supplementary Fig. 7a) [43]. Beclin 1 depletion in Molm-13 cells, carried out using two Beclin 1 shRNAs (shBECN1#5 and shBECN1#7), enhanced Smac mimetic- and Z-VAD-FMK-induced necroptosis (Fig. 8a and Supplementary Fig. 7b–f). The progress of the necroptosis was suppressed by GSK’963 and RIPK3 depletion (Fig. 8a and Supplementary Fig. 7b–h). In addition to Z-VAD-FMK, Beclin 1-depleted Molm-13 cells were also sensitised after exposure to emricasan, another caspase inhibitor (Fig. 8b and Supplementary Fig. 7b–f). As a result of necroptotic stimuli, an increase in MLKL oligomerisation was observed in this cell line, indicating that Beclin 1 also functions as an anti-necroptotic factor by suppressing MLKL oligomerisation in the AML cell line (Fig. 8c, d). To validate the anti-necroptotic role of Beclin 1 in vivo, a skin xenograft analysis was performed. The results showed that treatment with birinapant plus emricasan in shGFP-expressing groups suppressed the growth of tumour by up to 20%. While Beclin 1 depletion alone did not have observable effects on tumour growth compared to those of shGFP in the vehicle group, treatment with birinapant and emricasan induced a 50.3% reduction in the growth of the tumour (Fig. 8e–i). Furthermore, RIPK3 depletion reversed the effect of Beclin 1 depletion on tumour growth, which was induced by treatment with birinapant and emricasan, suggesting that depletion of Beclin 1 might be accelerating the necroptotic processes in the tumour model (Fig. 8e–i and Supplementary Fig. 7i). Collectively, these results indicate that Beclin 1 down-regulation renders the AML cells sensitive to necroptotic cell death, implying that Beclin 1 can be used as a plausible therapeutic target for necroptosis-induced anti-tumour therapy.

## Discussion

Necroptosis is a potent form of regulated cell death, which could induce the uncontrolled systemic induction of immune responses. Thus, several checkpoint regulators may be necessary to block the uninhibited progress of necroptosis. Recently, several proteins modulating necroptosis have been reported [17–27]. These regulatory proteins are limited in the pre-necrosome complex step, which consists of the process before the formation of the RIPK1/RIPK3 complex. By suppressing the early necroptotic phase, the cells affected by cell death signals could avoid facing an irreversible cell death process. However, the presence of negative regulators for the late necroptotic phase involving the post-necrosome complex could not be excluded to delay the cell death process and alleviate its effects. In this study, we demonstrated that Beclin 1 functions as a negative regulator of necroptosis by its integration into the necrosome complex of phosphorylated RIPK1/RIPK3/MLKL. As such, Beclin 1 could be defined as a key regulator of the post-necrosome complex. The incorporation of Beclin 1 into the necrosome complex is interesting in that the process neither hinders the phosphorylation nor the complex formation process of RIPK1, RIPK3, and MLKL. Beclin 1 was found to be incorporated into the necrosome complex via MLKL interaction and inhibited necroptosis via blocking MLKL oligomerisation. The association between Beclin 1 and phosphorylated MLKL is further demonstrated via the confocal analyses showing that, under necroptotic stimuli, Beclin 1 and phosphorylated MLKL was co-localised in the cytoplasmic area of cells. This association in the cells was disrupted in the presence of NSA or GSK’872, an inhibitor of MLKL or RIPK3, respectively, further demonstrating the association of Beclin 1 with MLKL.

Incorporation of Beclin 1 is important to have a negative effect on necroptosis. When MLKL is phosphorylated in the pseudokinase domain by RIPK3, the intramolecular interaction between CCDs and pseudokinase domain is disrupted, leading to the formation of an extended conformation, producing an MLKL molecule with CCDs prepared to form multiple complexes of MLKL [8, 9, 11, 12, 38–41, 45, 46]. These MLKL complexes are known to translocate to the edge of a cell, causing membrane perforations and thus disruption of the plasma membrane [8, 9, 12, 13, 37]. Noteworthy, the CCD of Beclin 1 was found to interact with the CCDs of MLKL. Beclin 1 devoid of CCD was unable to suppress necroptosis when overexpressed, indicating that the CCD of Beclin 1 is important for its interaction with MLKL. Furthermore, NSA, a chemical which binds to the CCD region of MLKL to prevent its oligomerisation, also inhibited the binding of MLKL and Beclin 1 under necroptosis. The competition of NSA and Beclin 1 for MLKL CCDs indirectly indicates that Beclin 1 specifically binds to the MLKL CCD region. The binding of the CCD of Beclin 1 to the exposed CCDs of phosphorylated MLKL leads to the inhibition of MLKL multiplex formation, thereby significantly hindering the necroptosis process induced by MLKL oligomerisation (Fig. 7e). Of note, TAM kinase was shown to promote necroptosis by inducing phosphorylation at MLKL tyrosine 376 residue, inducing oligomerisation of the pseudokinase domain of MLKL and plasma membrane rupture [47]. Since Beclin 1 does not interact with the kinase domain of MLKL, further studies are required to identify how Beclin 1 could prevent MLKL oligomerisation possibly by affecting TAM kinase-dependent MLKL oligomerisation.

While our study demonstrated the association between Beclin 1 and MLKL and elucidated the mechanisms involved in the necroptosis process, further questions remain. Beclin 1 can function in both autophagy-dependent and -independent pathways. As shown in Fig. 1, the inhibition of necroptosis under conditions inducing autophagy and the interruption of this process by the ablation of Beclin 1 suggests the involvement of the autophagy pathway in necroptosis. This pathway and the role of Beclin 1 in this process need to be studied further. Here, we showed that Beclin 1 also functions in an autophagy-independent way by targeting MLKL oligomerisation under non-autophagic conditions. These observations, however, indicate the possibility of more intricate novel pathways, as Beclin 1 has multiple binding partners with its diverse protein–protein-binding domains, including BH3, CCD, and ECD (Supplementary Fig. 6). Since many proteins are known to regulate the function of Beclin 1 posttranslationally through phosphorylation, ubiquitination, ISGylation, and acetylation, the next step would be to determine whether these modulations of Beclin 1 affect the interaction between Beclin 1 and MLKL, which could lead to the discovery of unanticipated and novel pathways affecting necroptosis [29, 30]. Finally, we analysed the anti-necroptotic effect of Beclin 1 using a xenograft model system (Fig. 8). Beclin 1-depleted Molm-13 cells were sensitised to Smac mimetic and caspase inhibitor-mediated necroptosis in vitro and in vivo, which was recovered by RIPK3 inhibition. The anti-necroptotic effect of Beclin 1 in AML cells appears to arise from the ability to suppress MLKL oligomerisation, which in turn protects the AML cell from necroptosis. However, further studies on the suitability of Beclin 1 as an anti-tumour target would be required to develop a novel therapeutic target in anti-tumour therapy involving necroptosis.

In this study, we demonstrated that Beclin 1 is a new negative member of the necrosome complex. Beclin 1 interacts with the CCD of MLKL during necroptosis and consequently suppresses necroptosis by reducing MLKL oligomerisation. The autophagy-independent function of Beclin 1 in necroptosis provides an insight into the molecular mechanism of cell death and has therapeutic implications in necroptosis-related diseases and anti-tumour therapies.

## Supplementary information

supplementary Figure legends

Supplementary Figure 1

Supplementary Figure 2

Supplementary Figure 3

Supplementary Figure 4

Supplementary Figure 5

Supplementary Figure 6

Supplementary Figure 7
