# Supplementary material for: Beclin 1 functions as a negative modulator of MLKL oligomerisation by integrating into the necrosome complex
Source: Cell Death Differ. 2020 May 26;27(11):3065–81. doi: 10.1038/s41418-020-0561-9 (PMC7560833; doi:10.1038/s41418-020-0561-9)
Supplement: Supplementary file 1 — supplementary Figure legends [file 41418_2020_561_MOESM1_ESM.docx]

**Supplementary Figure Legends**

**Supplementary Figure 1. Beclin 1 depletion promotes necroptosis.**

(a-c) HT-29 (a), TC-1 (b), and L929 cells (c) transfected with the indicated siRNAs were treated with TBZ or TZ in the presence or absence of Nec-1 (TBZN or TZN). After treatment, the cells were stained with annexin V-FITC and 7-AAD (a, b) or annexin V-FITC alone (c) for flow cytometry. (d-f) HT-29 (d), TC-1 (e), and L929 (f) cells transfected with the indicated siRNAs were treated with TBZ (d, e) or TZ (f) under normal conditions or autophagic conditions induced by pre-incubation with HBSS media. After treatment, the cells were stained with annexin V-FITC and 7-AAD for flow cytometry. The knockdown efficiency of each protein targeted by siRNAs and autophagy marker, p62, was determined by immunoblotting.

**Supplementary Figure 2. Beclin 1 does not affect on the NF-κB activation.**

(a) HT-29, TC-1, and L929 cells were transfected with the indicated siRNAs for 48 h. After transfection, necroptosis-related proteins levels were determined by immunoblotting in each cell lysate. (b) HT-29 cells transfected with the indicated siRNAs were treated with TNFα for the indicated times. After treatment, the cells were analysed by immunoblotting.

**Supplementary Figure 3. Myeloid-specific Beclin 1 knockout mice.**

Genomic DNA was prepared via phenol-chloroform extraction. The genotypes were determined via PCR using specific primers for each gene.

**Supplementary Figure 4. Beclin 1 ablation increases cell death induced via necroptotic stimulus.**

(a) Beclin 1 wild type (WT) or knockout (KO) HT-29 cells were treated with TBZ in the presence or absence of GSK’963 (TBZG) for 4 h. After treatment, the cells were stained with annexin V-FITC and 7-AAD for flow cytometry. (b) Beclin 1 WT or KO HT-29 cells were treated with TBZ in the presence or absence of GSK’963 for 4 h. After treatment, the cells were lysed under non-reducing conditions for MLKL oligomerisationor reducing conditions, and then analysed by immunoblotting.

**Supplementary Figure 5. Beclin 1 reconstitution in Beclin 1 knockout cells reverses the increase in MLKL oligomerisation.**

(a-c) Beclin 1 WT and KO HT-29 cells were infected with retroviruses containing Beclin 1 WT-expressing plasmid for 48 h. (a) The protein levels were determined by immunoblotting. (b) To analyse cell death, the cells were treated with TBZ in the absence or presence of GSK’963 for 4 h, and then stained with annexin V-FITC and 7-AAD. The necroptotic population was analysed by flow cytometry. Data are represented as the mean ± S.D., n = 3, with **P < 0.01 and ***P < 0.001 at each point compared to the indicated graph with the two-sided Student’s t-test. (c) To analyse MLKL oligomerisation, the cells were treated with TBZ in the absence or presence of GSK’963 for 4 h, and then lysed under non-reducing conditions for MLKL oligomerisation or under reducing conditions, before analysis by immunoblotting. (d-g) HT-29 cells were first infected with lentiviruses containing shGFP or shBECN1, and then subsequently infected with retroviruses containing FLAG-BECN1 resistant to shBECN1#5 or control plasmid. (d) The protein levels were determined by immunoblotting. (e,f) To analyse cell death, the cells were treated with TBZ in the presence or absence of GSK'963 (TBZG) for 4 h. The cells were subsequently stained with annexin V-FITC and 7-AAD for flow cytometry analysis. Data are represented as the mean ± S.D., n = 3, with ns=non-significance, *P < 0.05, and ***P < 0.001 at each point compared to the indicated graph with the two-sided Student’s t-test. (g) HT-29 cells employed above were treated with TBZ for 5 h. The cells were subsequently fixed and stained using anti-Beclin 1 and p-MLKL antibodies, and DAPI and subjected to confocal analyses. The boxed areas are shown at higher magnification below. Scale bars = 20 μm.

**Supplementary Figure 6. Two coiled-coil domains of MLKL redundantly function in the interaction with Beclin 1.**

(a) Mapping of the binding affinity between each Beclin 1 domain and MLKL. (b) 293T cells transfected as indicated were immunoprecipitated using the anti-HA antibody, then analysed by immunoblotting. (c) Mapping of the binding affinity between each MLKL domain and Beclin 1. (d-e) 293T cells transfected as the indicated plasmids were immunoprecipitated using the anti-HA or FLAG antibodies and analysed by immunoblotting.

**Supplementary Figure 7. Molm-13 expressing shBECN1 are sensitised upon treatment with necroptotic stimuli.**

(a) Molm-13 cells were treated with 0.05 μM birinapant (Biri) and 20 μM Z-VAD-FMK or 1 μM emricasan (Emri) in the presence or absence of 2 μg/mL anti-TNF antibody for 12 h, stained with propidium iodide (PI), and then subjected to flow cytometry. Data are represented as the mean ± S.D., n = 3, with **P < 0.01 and ***P < 0.001 at each point compared to the indicated graph using a two-sided Student’s t-test. (b) Molm-13 cells were infected with lentivirus containing shGFP or shBECN1#5, and the protein levels were determined by immunoblotting. (c) Representative flow cytometry plot images for Figure 8a, b. (d) Molm-13 cells were infected with lentivirus containing shGFP or shBECN1#7, and the protein levels were determined by immunoblotting. (e, f) Molm-13 cells expressing GFP shRNA or Beclin 1 shRNA #7 were treated with 0.05 μM birinapant (Biri) and 20 μM Z-VAD-FMK or 1 μM emricasan (Emri) in the presence or absence of 100 nM GSK'963 for 10 h, stained with propidium iodide (PI), and then subjected to flow cytometry. Data are represented as the mean ± S.D., n = 3, with **P < 0.01 and ***P < 0.001 at each point compared to the indicated graph using a two-sided Student’s t-test. (g, h) Molm-13 cells were infected with lentivirus containing shGFP, shBECN1#5, or shBECN1#5 plus shRIPK3. (g) The protein levels were determined by immunoblotting. (h) Molm-13 cells expressing indicated shRNAs were treated with 0.05 μM birinapant (Biri) and 20 μM Z-VAD-FMK or 1 μM emricasan (Emri) for 10 h, stained with propidium iodide (PI), and then subjected to flow cytometry. Data are represented as the mean ± S.D., n = 3, with **P < 0.01 and ***P < 0.001 at each point compared to the indicated graph using a two-sided Student’s t-test. (i) After 18 days of tumour inoculation, the protein levels in the resected tumours were analysed by immunoblotting.
