# Supplementary figures and images for: Beclin 1 functions as a negative modulator of MLKL oligomerisation by integrating into the necrosome complex
Source: Cell Death Differ. 2020 May 26;27(11):3065–81. doi: 10.1038/s41418-020-0561-9 (PMC7560833; doi:10.1038/s41418-020-0561-9)

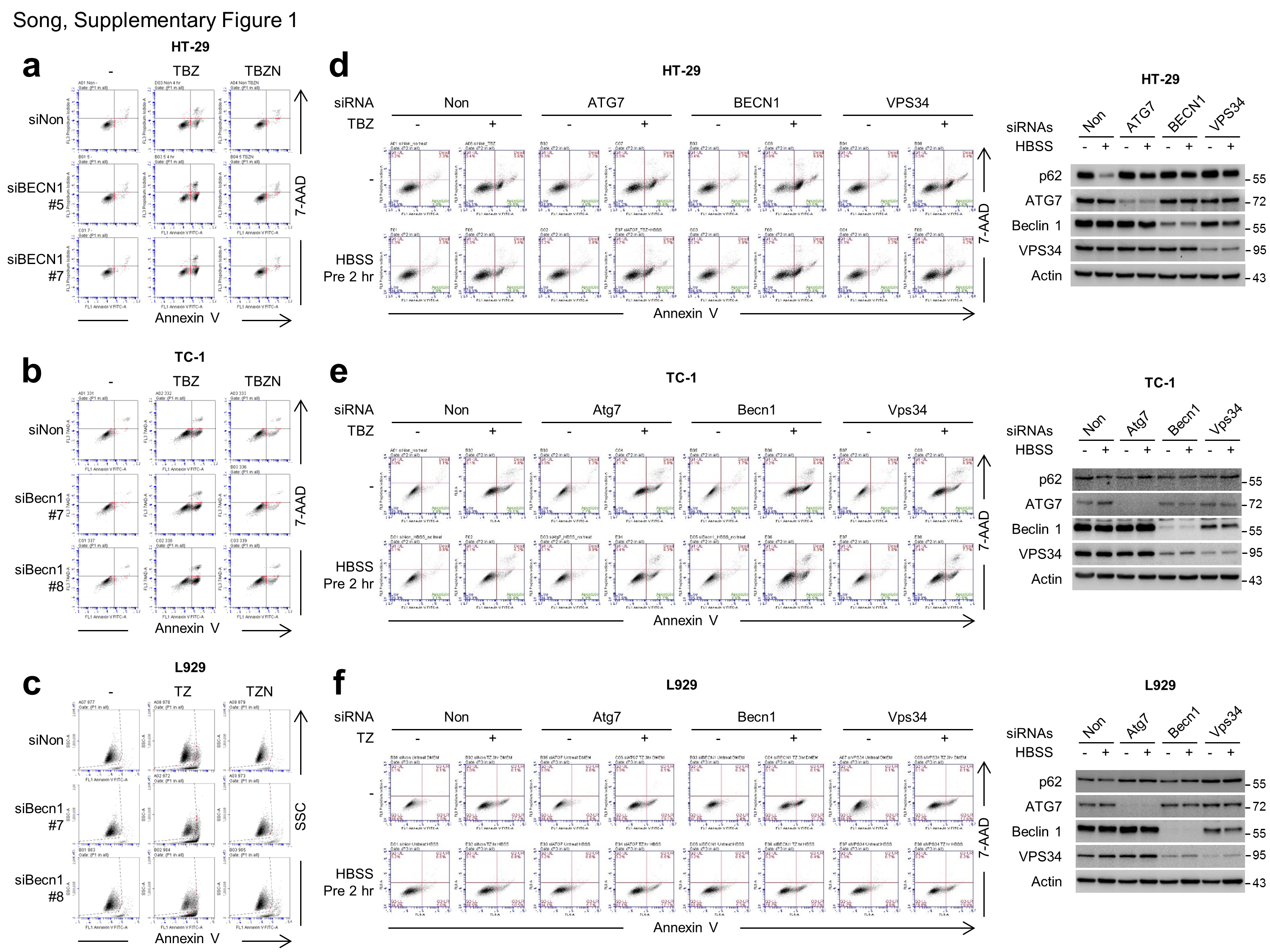

Supplement: Supplementary file 2 — Supplementary Figure 1 [file 41418_2020_561_MOESM2_ESM.tif]

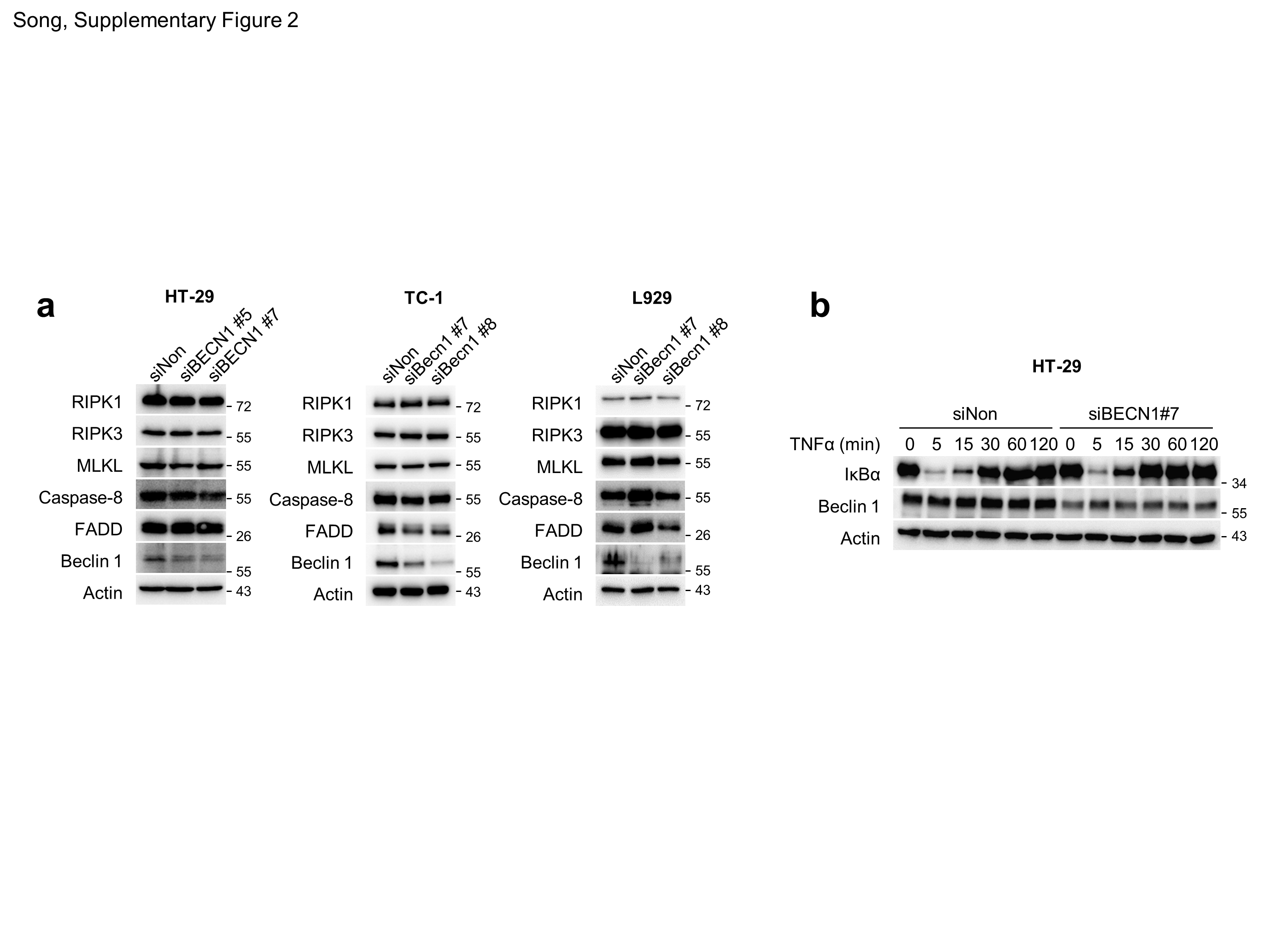

Supplement: Supplementary file 3 — Supplementary Figure 2 [file 41418_2020_561_MOESM3_ESM.tif]

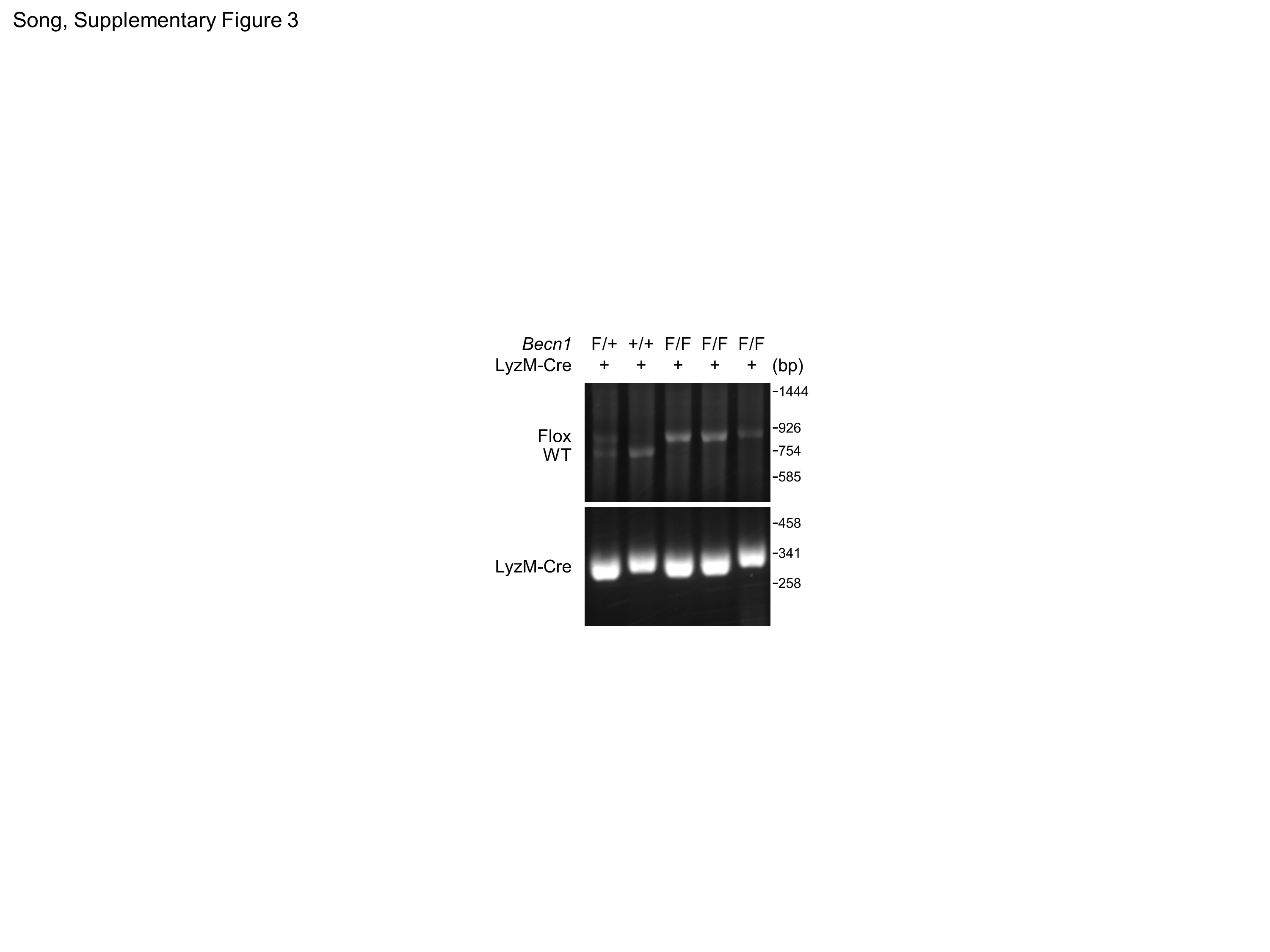

Supplement: Supplementary file 4 — Supplementary Figure 3 [file 41418_2020_561_MOESM4_ESM.tif]

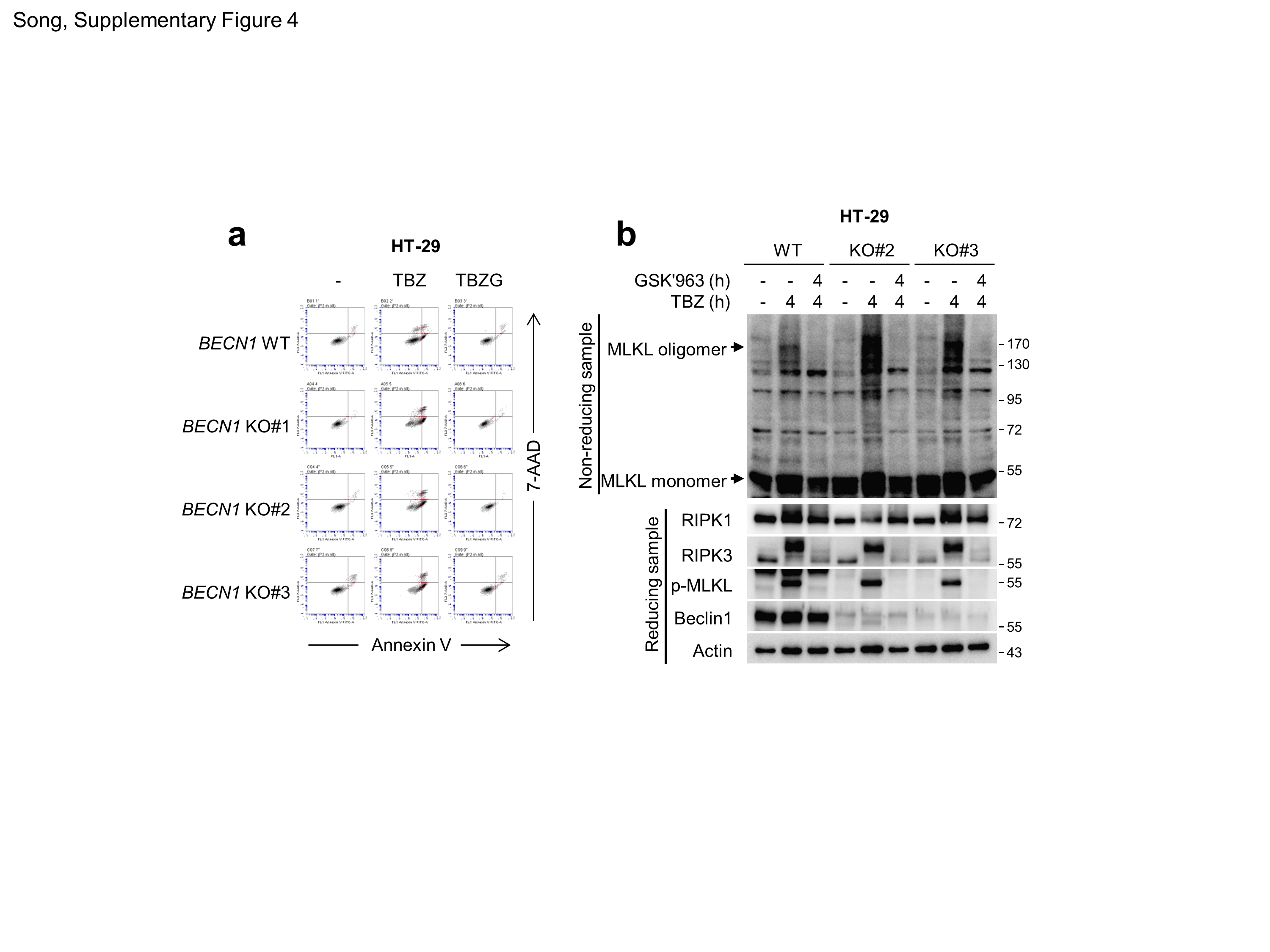

Supplement: Supplementary file 5 — Supplementary Figure 4 [file 41418_2020_561_MOESM5_ESM.tif]

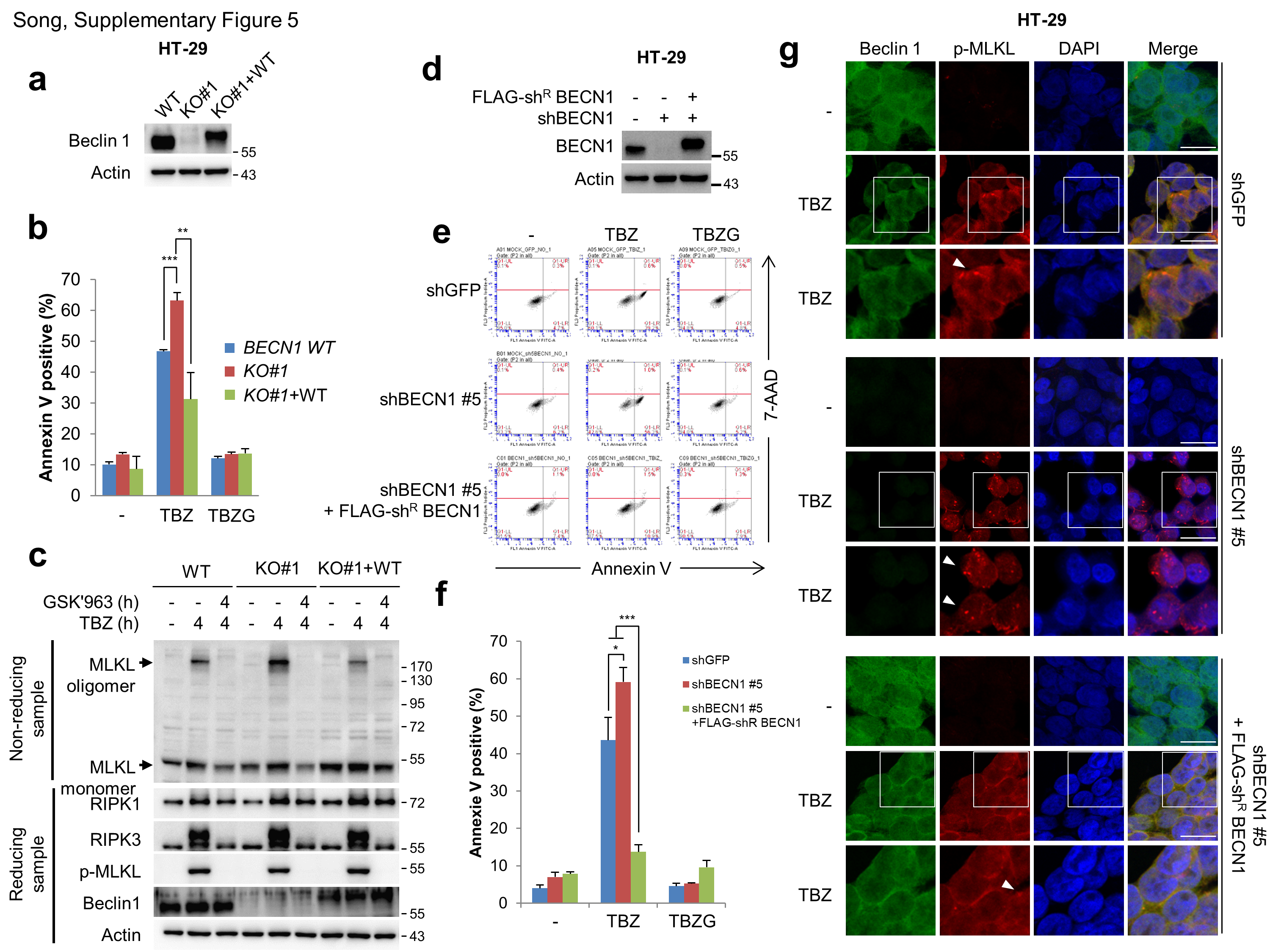

Supplement: Supplementary file 6 — Supplementary Figure 5 [file 41418_2020_561_MOESM6_ESM.tif]

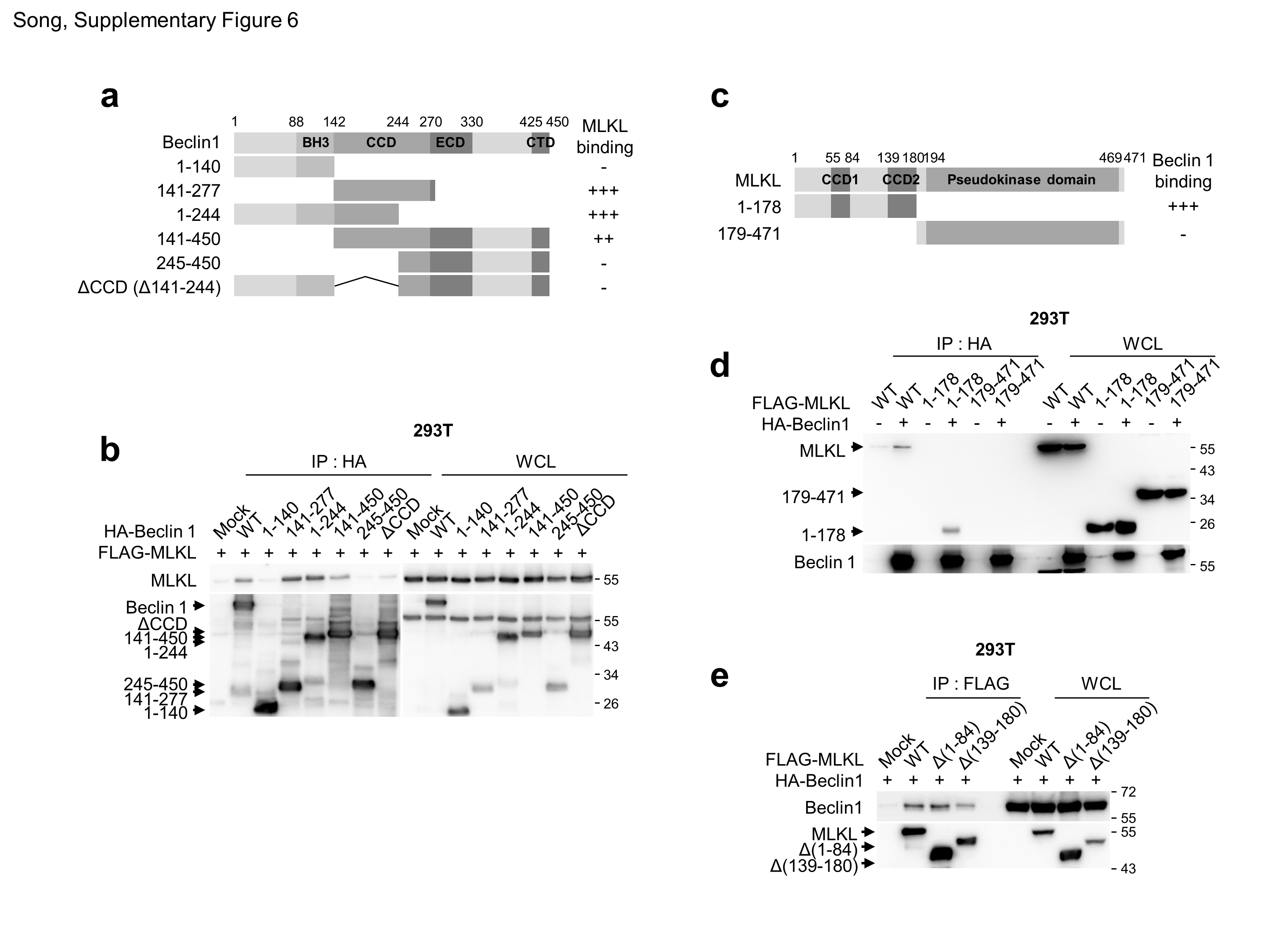

Supplement: Supplementary file 7 — Supplementary Figure 6 [file 41418_2020_561_MOESM7_ESM.tif]

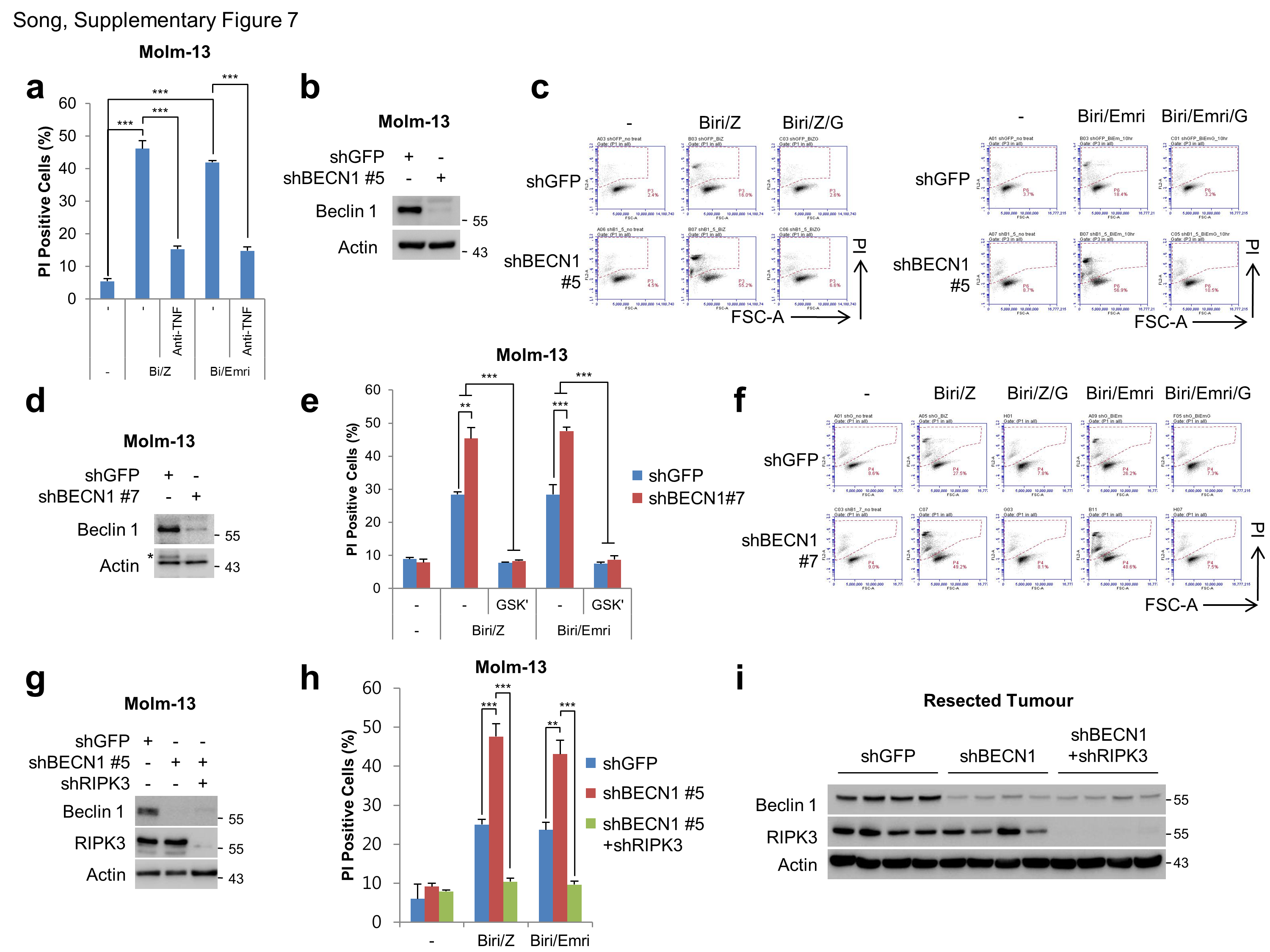

Supplement: Supplementary file 8 — Supplementary Figure 7 [file 41418_2020_561_MOESM8_ESM.tif]
